# Supplementary material for: The IL-33/ST2 axis is protective against acute inflammation during the course of periodontitis
Source: Nat Commun. 2024 Mar 28;15:2707. doi: 10.1038/s41467-024-46746-2 (PMC10978877; doi:10.1038/s41467-024-46746-2)
Supplement: Supplementary file 1 — Supplementary Information [file 41467_2024_46746_MOESM1_ESM.pdf]

# Supplementary Information

## The IL-33/ST2 axis is protective against acute inflammation during the course of periodontitis

Liu et al.

Supplementary Fig. 1

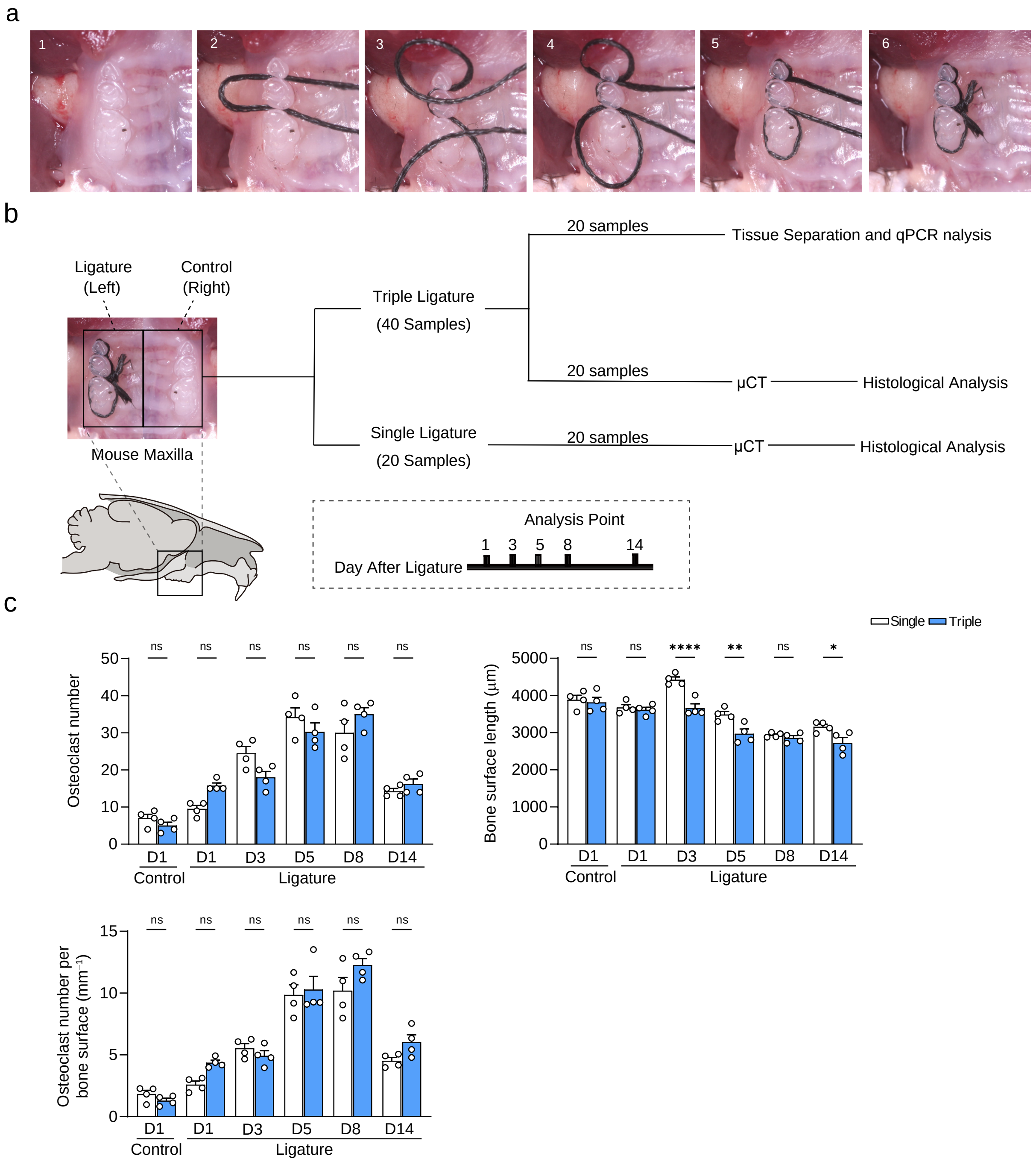


**Supplementary Fig. 1 The experiment design and induction of the modified ligature model. | a** Step-by-step instruction for the triple-ligature placement. More details are shown in Supplementary Movie. 1. **b** Experimental design and analysis time course. The mouse skull cartoon is created by Kennedy, A. Zenodo. DOI: 10.5281/zenodo.3925962. **c** Temporal changes in osteoclast number, bone surface length and osteoclast number per bone surface in the second molar region (*n* = 4 mice per group). D1: Day 1; D3: Day 3; D5: Day 5; D8: Day 8; D14: Day 14. Data are presented as the mean ± SEM. *P < 0.05; ns (not significant), P > 0.05; by two-way ANOVA with multiple comparisons via Šídák's method. The exact P-values are shown in Supplementary Data. 2. Source data are provided as a Source Data file.

Supplementary Fig. 2

a

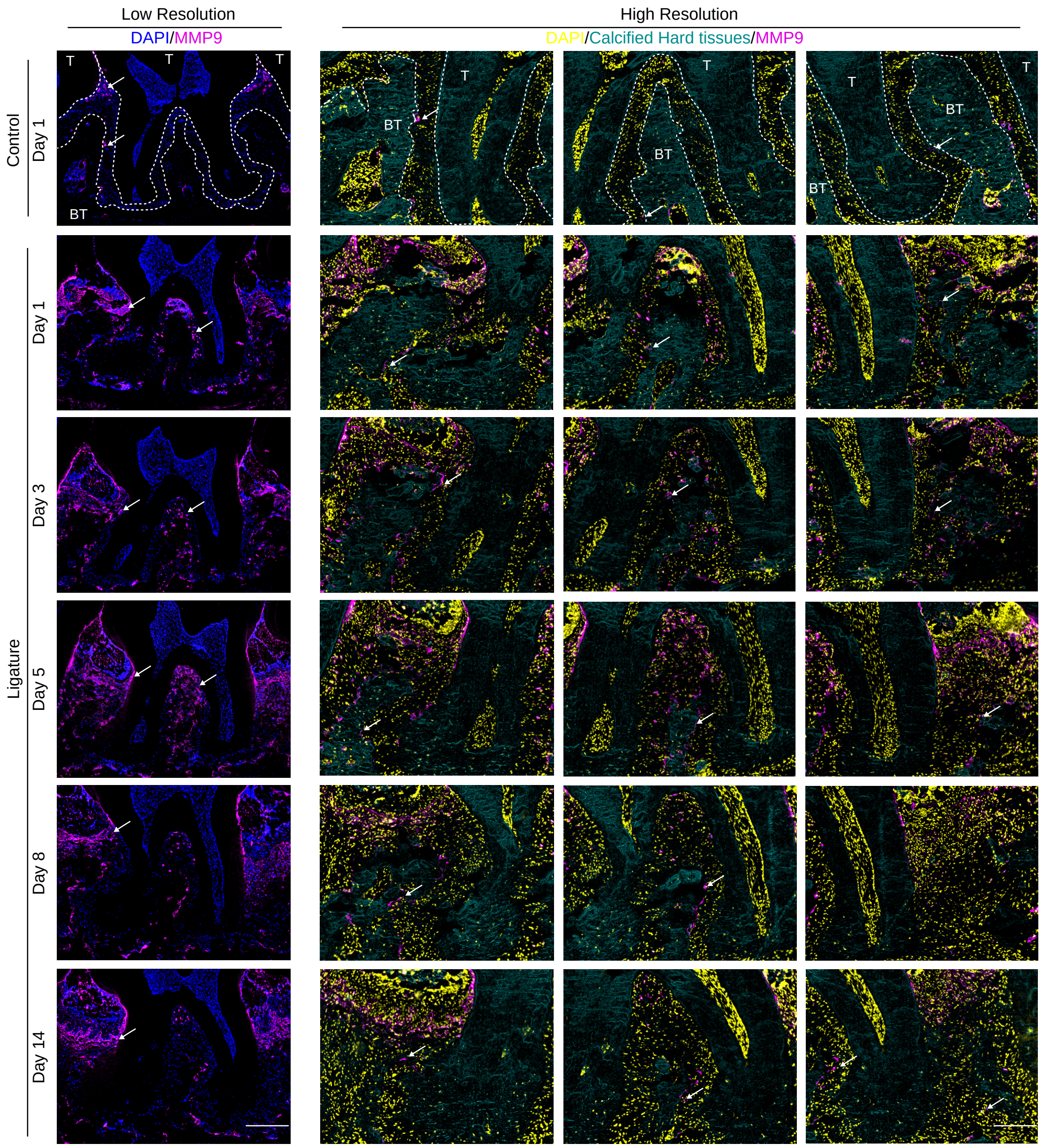


b

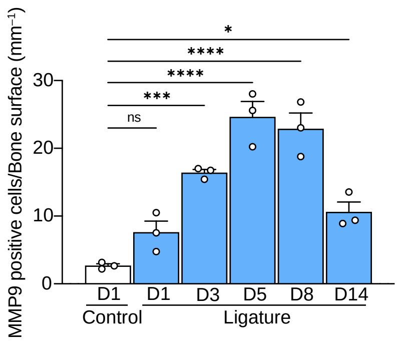

**Supplementary Fig. 2 The confirmation of TRAP staining via the immunofluorescence staining of MMP9. | a** Temporal change in MMP9 positive cells in the representative immunofluorescence staining images of three independent mouse experiments. Scale bar, 300 μm for low resolution and 60 μm for high resolution. **b** Temporal changes in MMP9 positive cell (attached on the bone surface) number per bone surface in the second molar region ($n$ = 3 mice per group). T, Tooth; BT, bone tissue. D1: Day 1; D3: Day 3; D5: Day 5; D8: Day 8; D14: Day 14. The white arrows indicate the MMP9 positive cells. Data are presented as the mean ± SEM. *P < 0.05; **P < 0.01; ***P < 0.001; ns (not significant), P > 0.05; by one-way ANOVA with multiple comparisons via Dunnett's test (**b**)**.** The exact P-values are shown in Supplementary Data. 2. Source data are provided as a Source Data file.

Supplementary Fig. 3

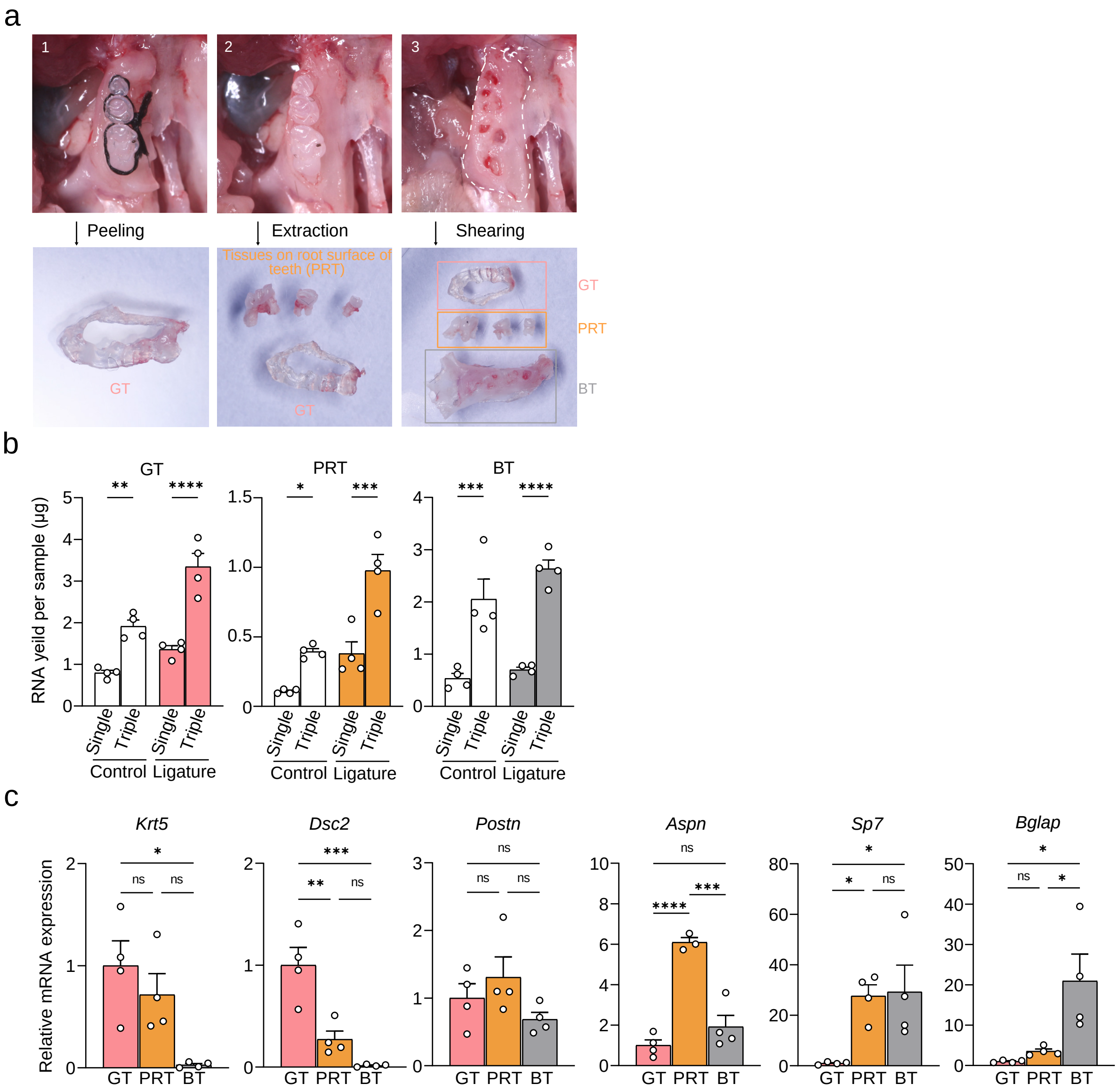


**Supplementary Fig. 3 Evaluation of the reliability of tissue separation. | a** Step-by-step instruction for tissue separation into GT, PRT, and BT. Separation is performed in the RNAlater or TRIzol in the actual experiments. **b** Total RNA yield from the different tissues in the two models on Day 8 ($n$ = 4 mice per group). GT: gingiva tissue, PRT: peri-root tissue, BT: bone tissue. **c** mRNA expression of tissue-specific genes on the control side of the three tissues (on Day 8; GT = 1; $n$ = 4 mice per group except for the group highlighted in Source Data file, which were $n$ = 3 mice). *Krt5* and *Dsc2* are for the verification of GT; *Postn* and *Aspn* are for PRT; *Sp7* and *Bglap* are for BT. Data are presented as the mean ± SEM. *P < 0.05; **P < 0.01; ***P < 0.001; ****P < 0.0001; ns (not significant), P > 0.05; by two-way ANOVA with multiple comparisons via Šídák's method (**b**); by one-way ANOVA with multiple comparisons via Tukey's test (**c**). The obvious outliers were evaluated and excluded by Grubb's test ($\alpha$ = 0.05). The exact P values are shown in Supplementary Data. 2. Source data are provided as a Source Data file.

Supplementary Fig. 4

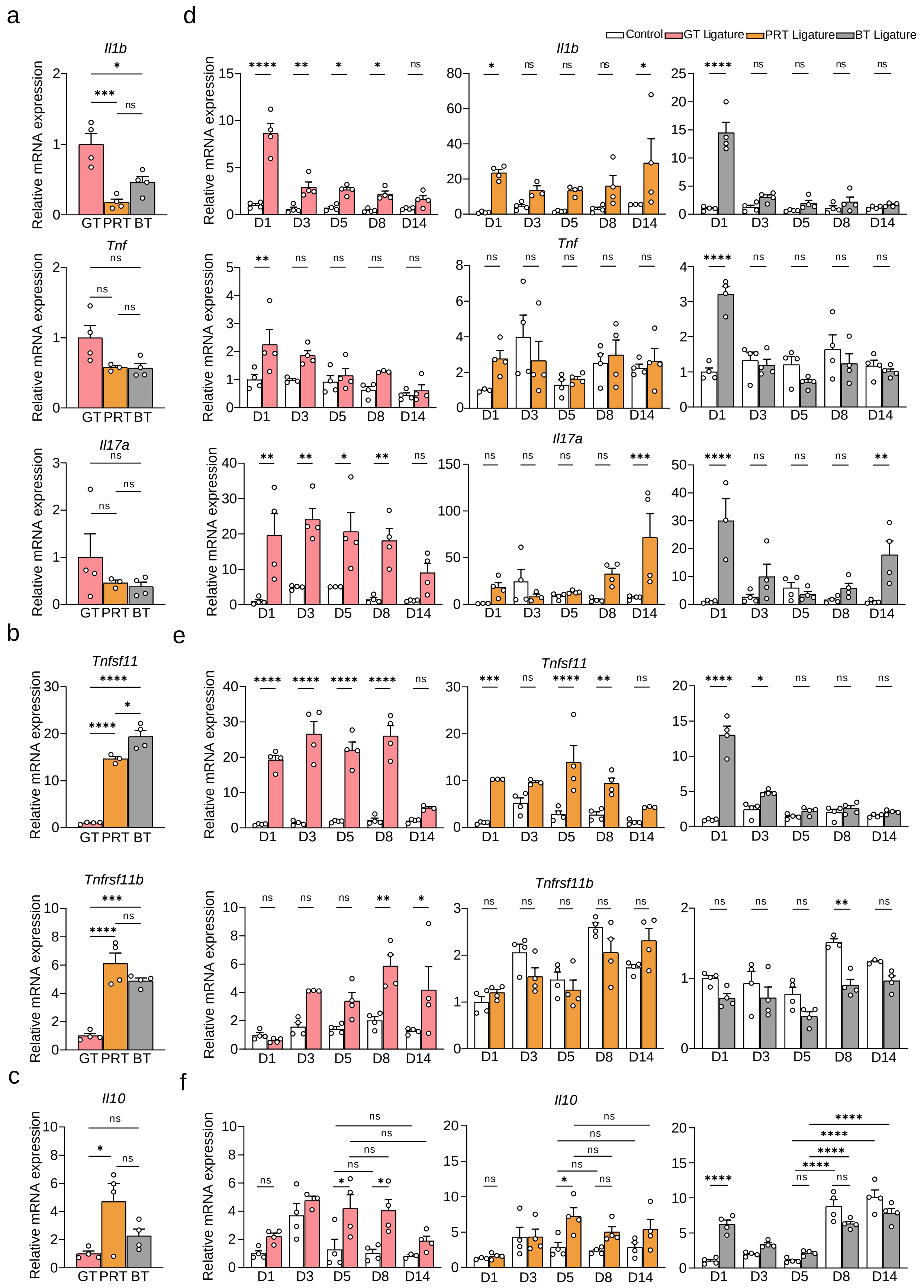

**Supplementary Fig. 4 Inflammation and osteoclastogenesis-related cytokines expression in three tissues. | a-c** mRNA expression of *Il1b*, *Tnf*, *Il17a* (**a**), *Tnfsf11*, *Tnfrsf11b* (**b**) and *Il10* (**c**) on the control side of the three tissues (on Day 1; GT = 1; *n* = 4 mice per group except for the group highlighted in Source Data file, which were *n* = 3 mice). **d-f** Temporal changes in mRNA expression of *Il1b*, *Tnf*, *Il17a* (**d**), *Tnfsf11*, *Tnfrsf11b* (**e**) and *Il10* (**f**) in the three tissues (Control Day 1 of each tissue = 1; *n* = 4 mice per group except for the group highlighted in Source Data file, which were *n* = 3 mice). GT: gingiva tissue, PRT: peri-root tissue, BT: bone tissue. D1: Day 1; D3: Day 3; D5: Day 5; D8: Day 8; D14: Day 14. Data are presented as the mean ± SEM. *P < 0.05; **P < 0.01; ***P < 0.001; ****P < 0.0001; ns (not significant), P > 0.05; by one-way ANOVA with multiple comparisons via Tukey's test (**a-c**); and two-way ANOVA with multiple comparisons via Šídák's method (**d-f**). The obvious outliers were evaluated and excluded by Grubb's test ($\alpha$ = 0.05). The exact P values are shown in Supplementary Data. 2. Source data are provided as a Source Data file.

Supplementary Fig. 5

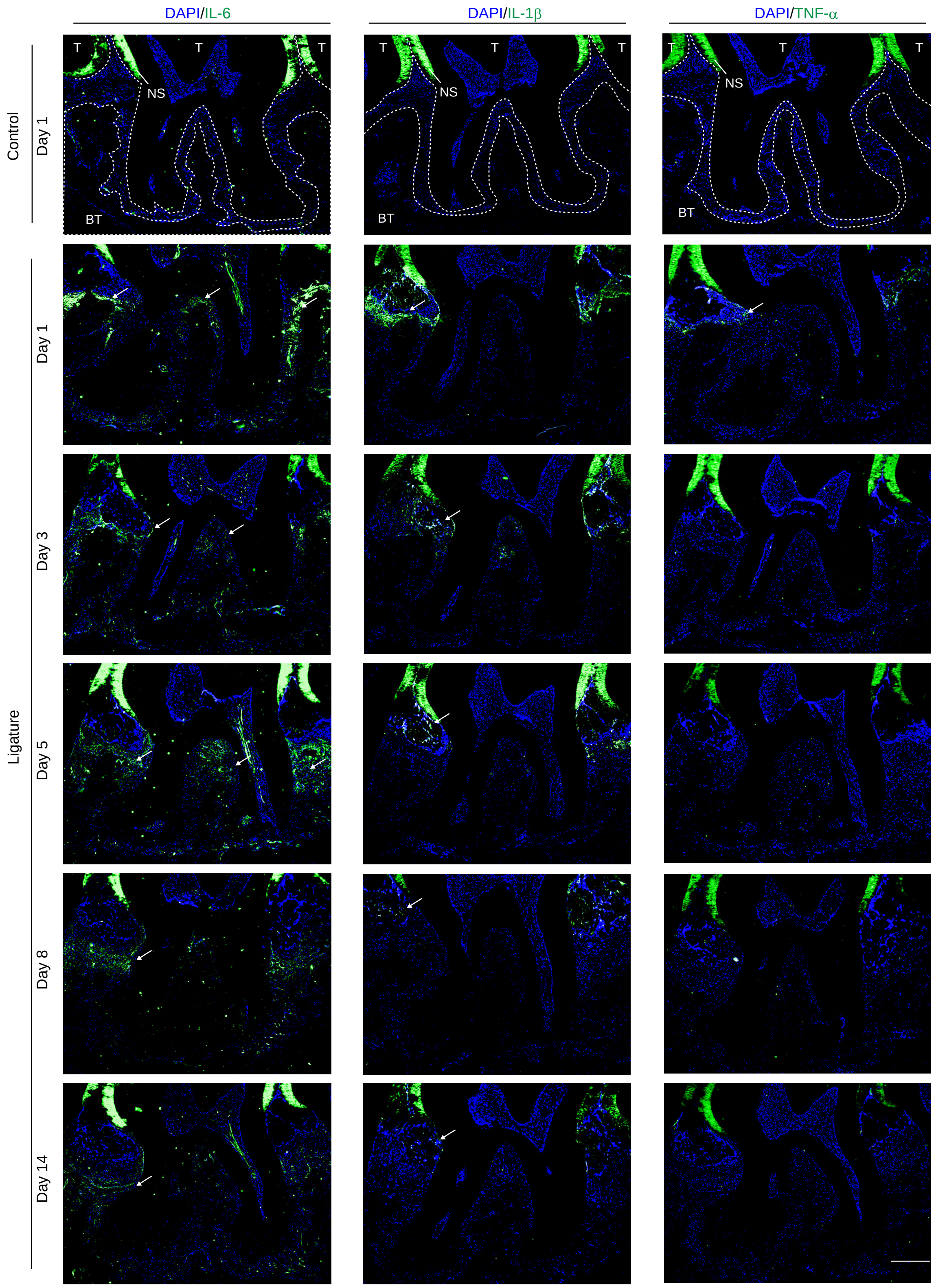


**Supplementary Fig. 5 Temporal changes in immunofluorescence staining of pro-inflammatory cytokines.** | Representative IL-6, IL-1β, and TNF-α immunofluorescence staining images of three independent mouse experiments. T, Tooth; NS, nonspecific signal; BT, bone tissue. Scale bar, 300 μm.

Supplementary Fig. 6

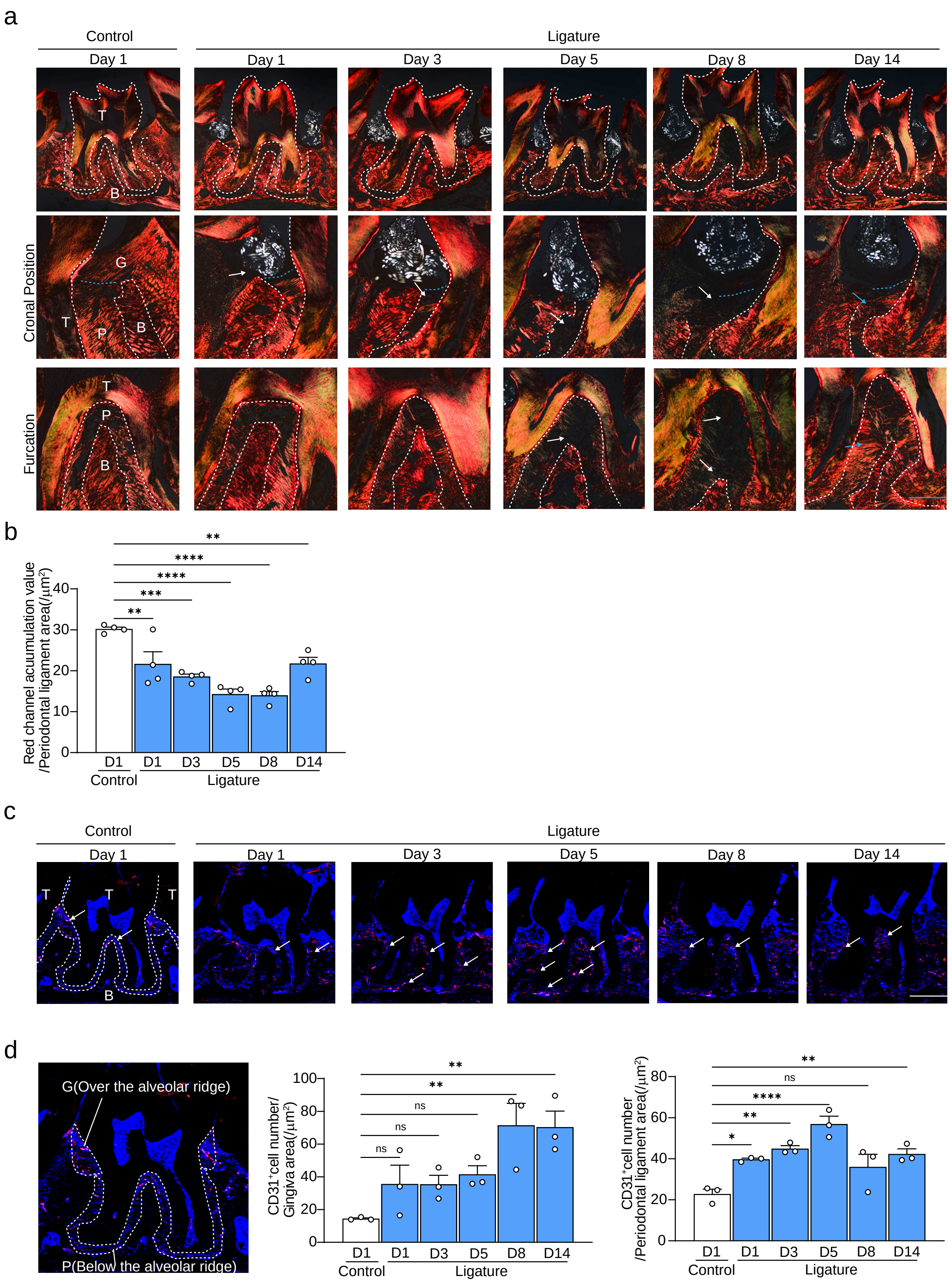

**Supplementary Fig. 6 Temporal histological analysis of the pathogenesis. | a** Representative images of Picro-Sirius Red staining in the second molar region (high resolution for coronal and furcation region) from four independent mouse experiments. The tissues are divided by white dotted lines into T, G, P, and B. The white arrows indicate the destruction of type I collagen fibers. The blue arrows indicate the regeneration of type I collagen fibers. T: tooth; G, Gingiva; P: periodontal ligament and connective tissue; B: alveolar bone; L: ligature. Scale bar, white one is 300 μm and blue one is 60 μm. **b** Temporal changes in red channel accumulation value per periodontal ligament area in the second molar region (*n* = 4 mice per group). D1: Day 1; D3: Day 3; D5: Day 5; D8: Day 8; D14: Day 14. **c** Representative images of CD31 immunofluorescence staining in the second molar region of three independent mouse experiments. The white arrows indicate the CD31 positive cells. Scale bar, 300 μm. **d** Temporal changes in $CD31^+$ cell number per gingival or periodontal ligament area in the second molar region (*n* = 4 mice per group). G, gingiva area; P, periodontal ligament area. Data are presented as the mean ± SEM. *P < 0.05; **P < 0.01; ***P < 0.001; ****P < 0.0001; ns (not significant), P > 0.05; by one-way ANOVA with multiple comparisons via Dunnett's test (**b**, **d**). The exact P values are shown in Supplementary Data. 2. Source data are provided as a Source Data file.

Supplementary Fig. 7

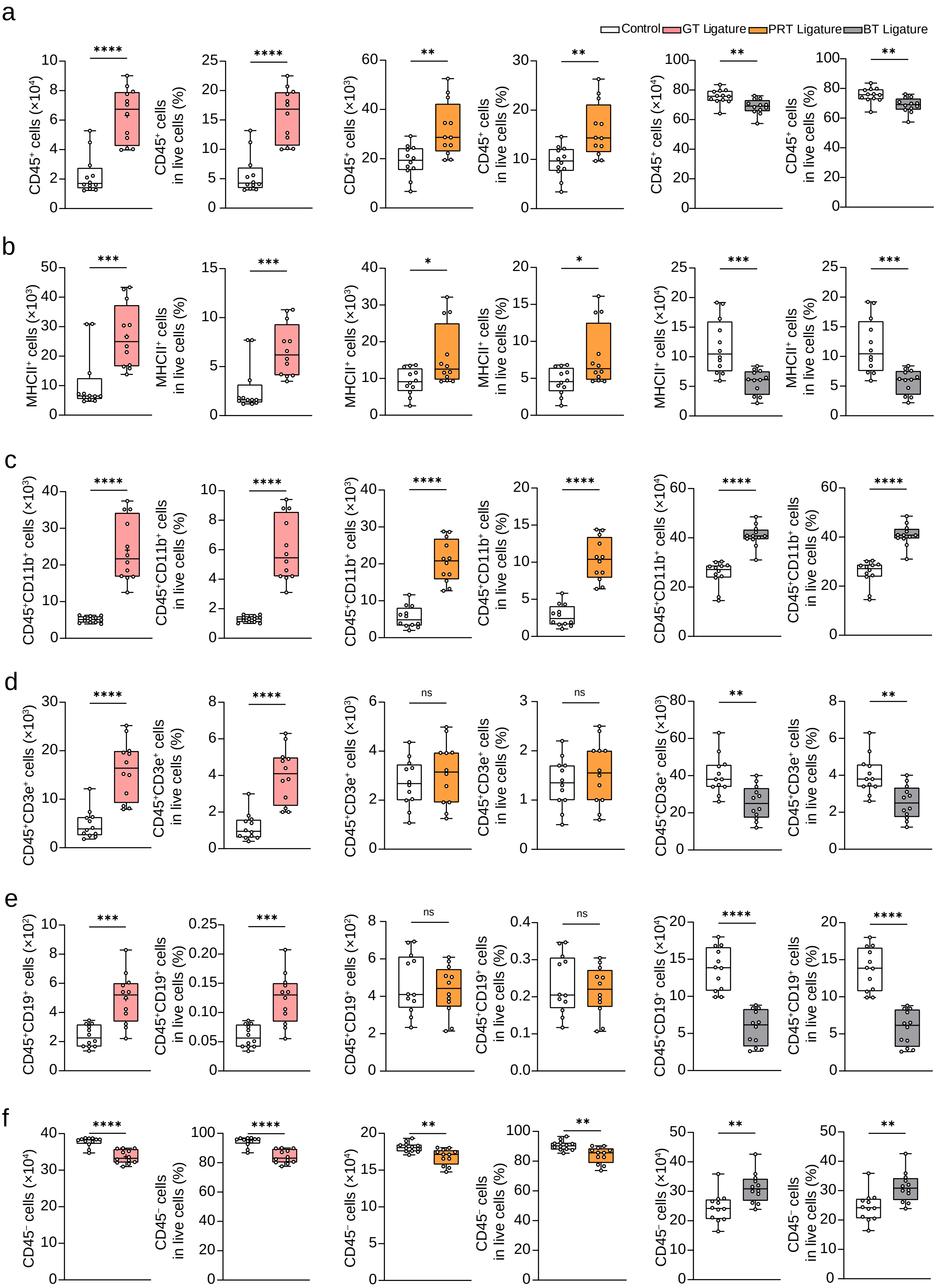


**Supplementary Fig. 7 Cell component alteration in three tissues on Day 5. | a** The number and percentages (in live cells) of CD45⁺ (**a**), MHCII⁺ (**b**), CD45⁺CD3ε⁺ (**c**), CD45⁺CD19⁺ (**d**), CD45⁺CD11b⁺ (**e**), and CD45⁻ (**f**) cells in three tissues on day 5 are shown horizontally by the tissue type (*n* = 12 mice per group). GT: gingiva tissue, PRT: peri-root tissue, BT: bone tissue. Data are presented as the mean ± SEM. *P < 0.05; **P < 0.01; ***P < 0.001; ****P < 0.0001; by two-side unpaired t-test with Welch's correction. The exact P values are shown in Supplementary Data. 2. The related gating strategy was shown in Supplementary Fig. 29. Source data are

Supplementary Fig. 8

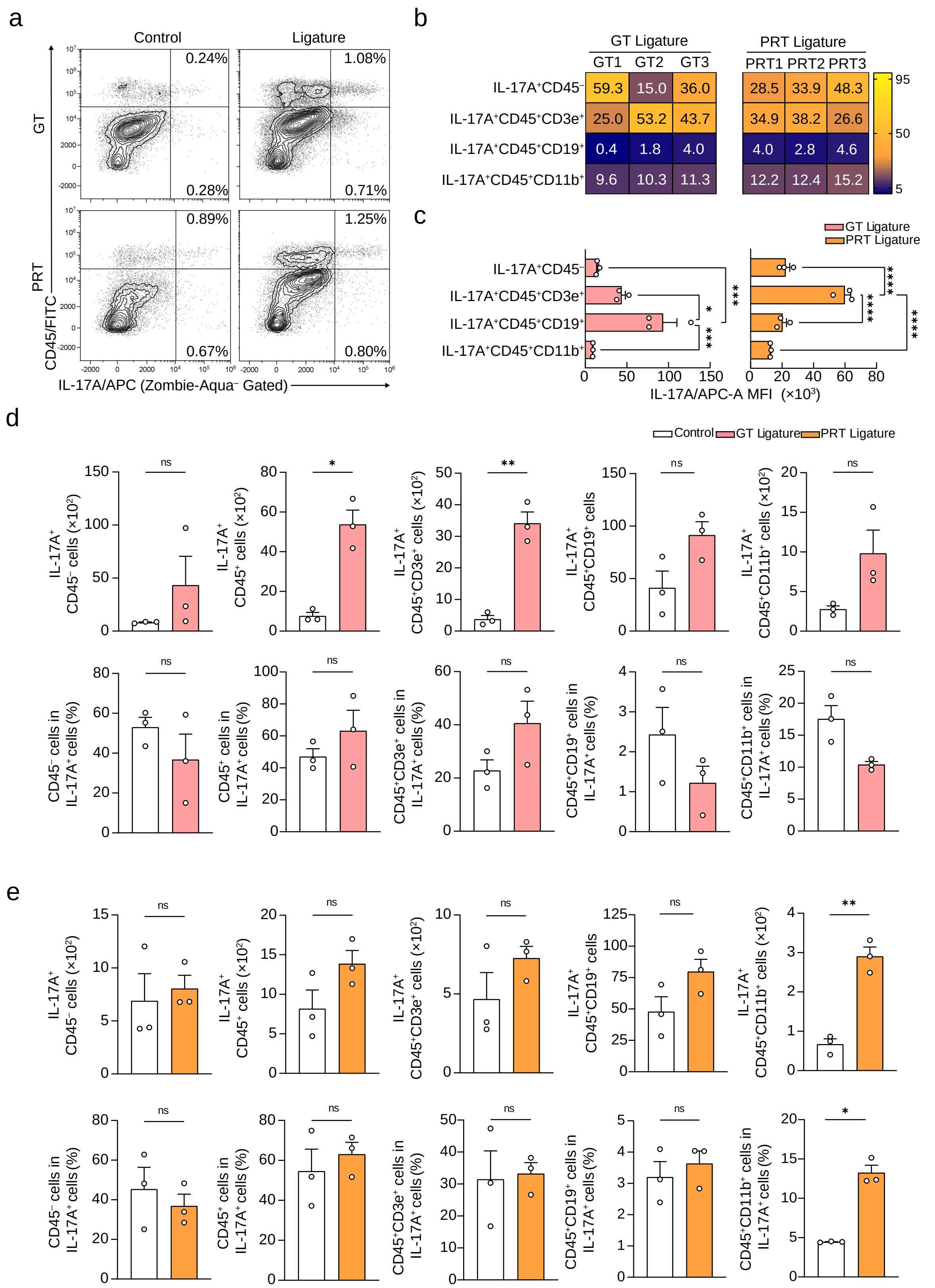

**Supplementary Fig. 8 Source of IL-17A in GT and PRT on Day 5. | a** Changes in IL-17A$^+$ cells in GT and PRT with or without ligature placement. A representative contour plot is shown, and the percentages shown in the gates are the mean of three individual mice experiments. GT: gingiva tissue, PRT: peri-root tissue. **b** Heat map of the percentage of different IL-17A$^+$ lineage in ligatured GT and PRT. Data from three independent experiments are shown (*n* = 3 mice per group, GT/PRT 1, 2, 3). **c** MFI (median fluorescence intensity) of the different IL-17A$^+$ lineages in ligatured GT and PRT (*n* = 3 mice per group). **d, e** Changes in the cell number and percentages (in IL-17A$^+$ or IL-17A$^+$CD45$^+$ cells) of different IL-17A$^+$ lineages in GT (**d**) and PRT (**e**) are shown perpendicularly (*n* = 3 mice per group). Data are presented as the mean ± SEM except for **c-e**. *P < 0.05; **P < 0.01; ***P < 0.001; ****P < 0.0001; ns (not significant), P > 0.05; by one-way ANOVA with multiple comparisons via Tukey's test (**c**); and two-side unpaired t-test with Welch's correction (**d, e**). The exact P values are shown in Supplementary Data. 2. The related gating strategy was shown in Supplementary Fig. 29. Source data are provided as a Source Data file.

Supplementary Fig.9

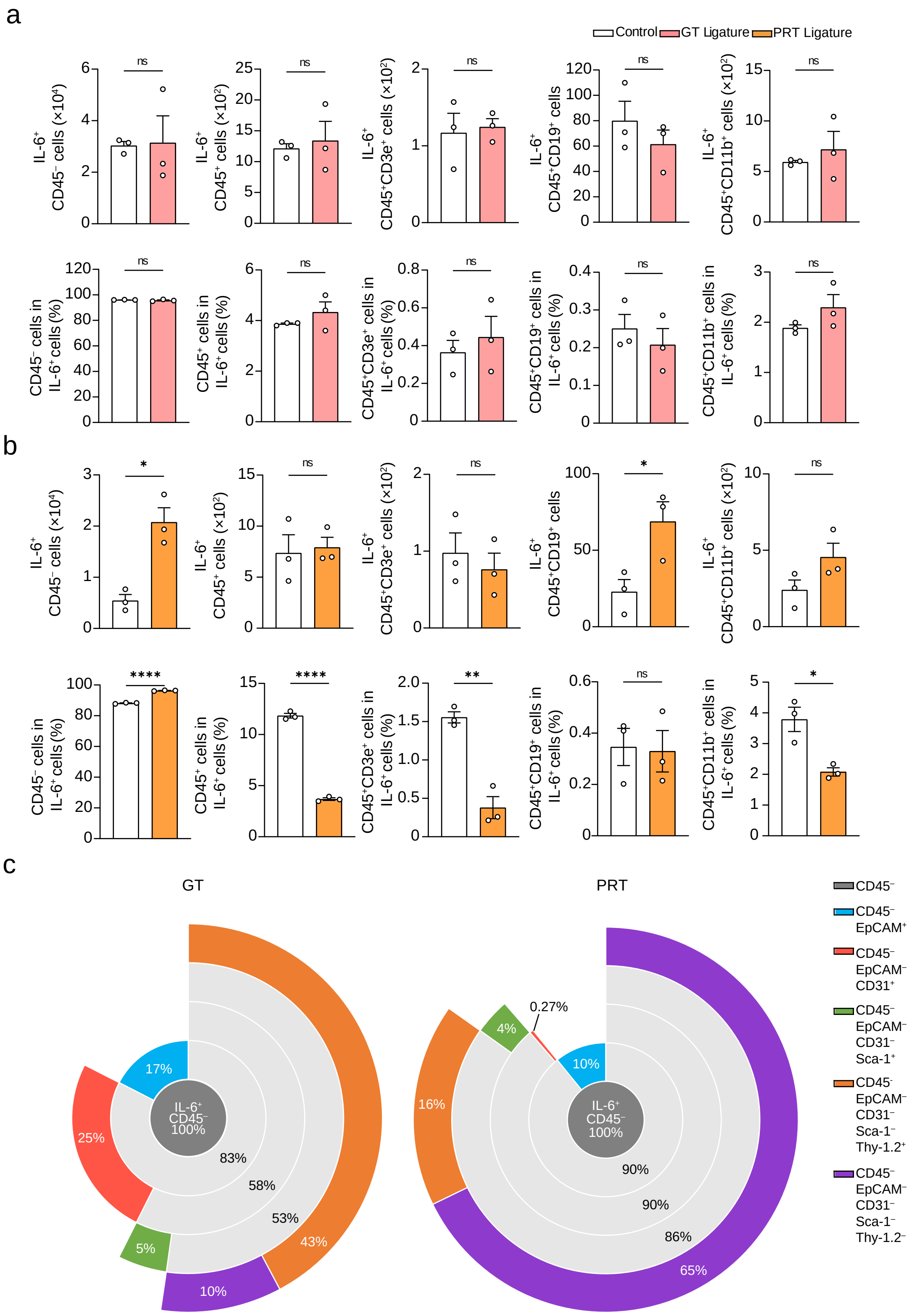


**Supplementary Fig. 9 Source of IL-6 in GT and PRT on Day 5. | a**, **b** Changes in the cell number and percentages (in IL-6$^+$ or IL-6$^+$CD45$^+$ cells) of different IL-6$^+$ lineages in GT (**a**) and PRT (**b**) are shown perpendicularly ($n$ = 3 mice per group). GT: gingiva tissue, PRT: peri-root tissue. **c** Sunburst showing the composition of IL-6$^+$CD45$^+$ cells in GT and PRT. The percentages shown in the populations are the mean of three individual-mice experiments. Data are presented as the mean ± SEM except for **c**. ns (not significant), P > 0.05; by two-side unpaired t-test with Welch's correction. The exact P values are shown in Supplementary Data. 2. The related gating strategy was shown in Supplementary Fig. 29 (**a, b**), 30 (**c**).Source data are provided as a Source Data file.

Supplementary Fig. 10

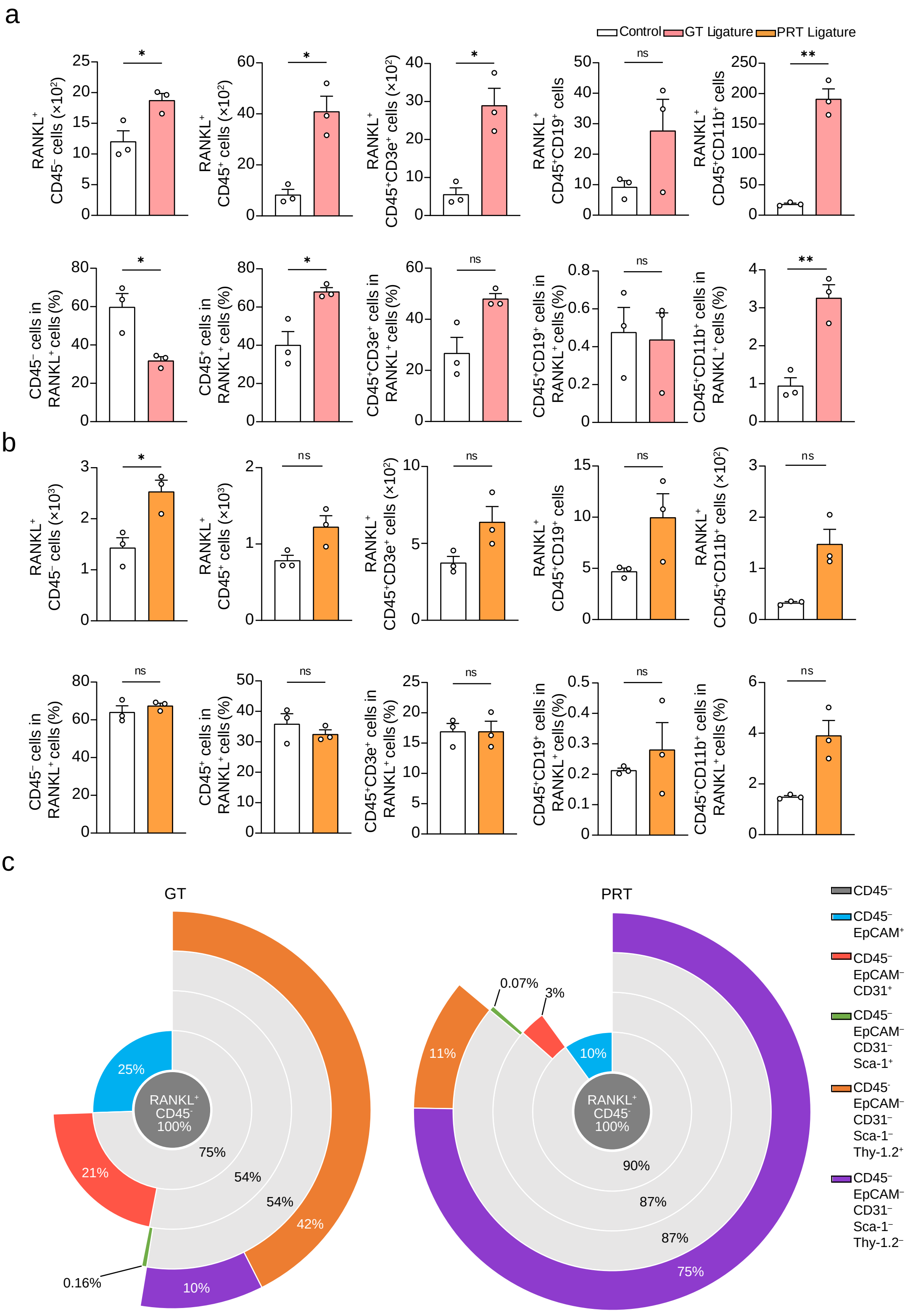


**Supplementary Fig. 10 Source of RANKL in GT and PRT on Day 5. | a, b** Changes in the cell number and percentages (in RANKL⁺ or RANKL⁺CD45⁺ cells) of different RANKL⁺ lineages in GT (**a**) and PRT (**b**) are shown perpendicularly (*n* = 3 mice per group). GT: gingiva tissue, PRT: peri-root tissue. **c** Sunburst showing the composition of RANKL⁺CD45⁻ cells in GT and PRT. The percentages shown in the populations are the mean of three individual-mice experiments. Data are presented as the mean ± SEM except for c. *P < 0.05; **P < 0.01; ns (not significant), P > 0.05; by two-side unpaired t-test with Welch's correction. The exact P values are shown in Supplementary Data. 2. The related gating strategy was shown in Supplementary Fig. 29 (**a, b**), 30 (**c**).Source data are provided as a Source Data file.

Supplementary Fig. 11

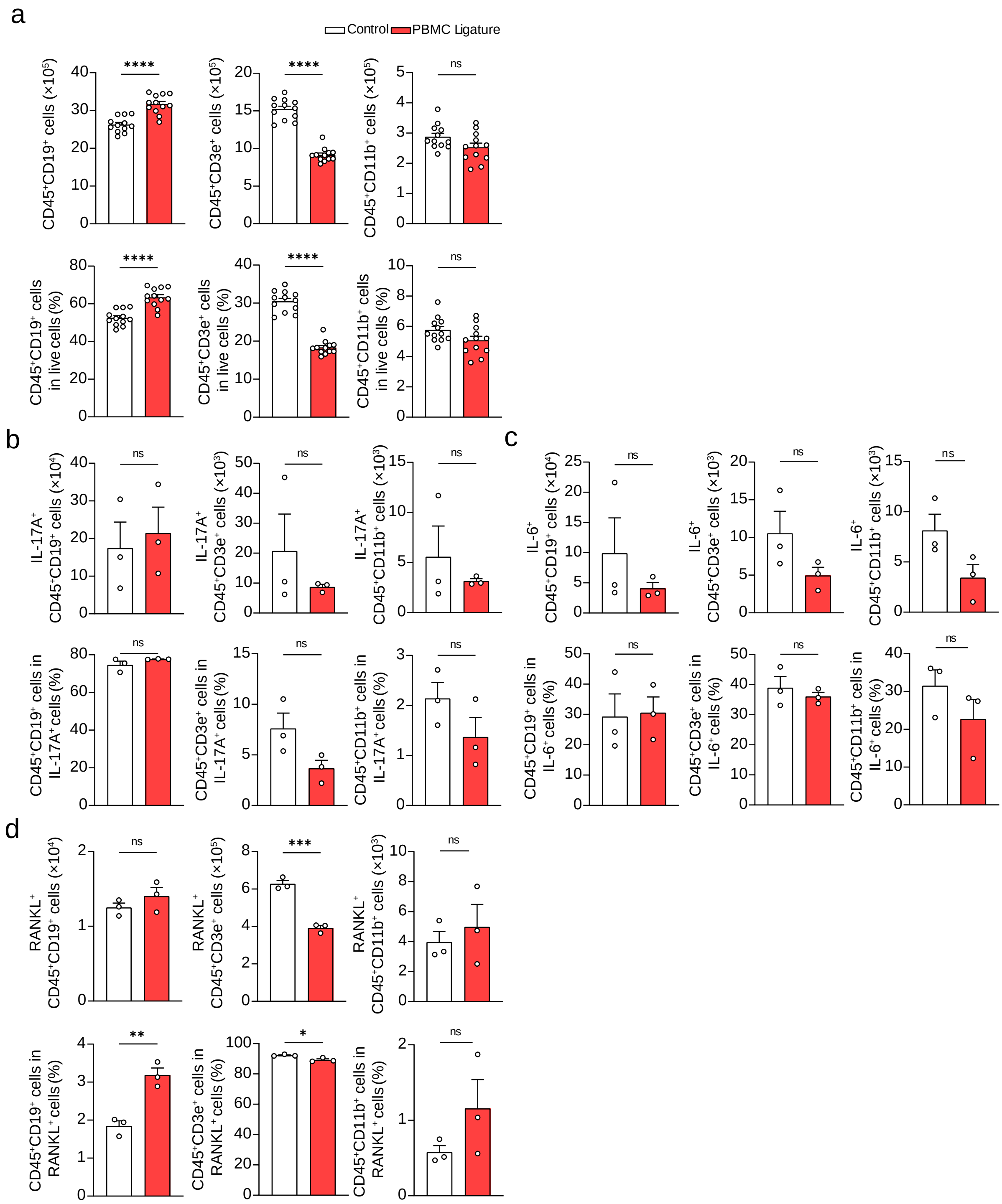

**Supplementary Fig. 11 Changes in the composition of PBMC and possible source of inflammation-related cytokines on Day 5. | a** Changes in the number and percentages (in live cells) of CD45⁺CD3ε⁺, CD45⁺CD19⁺, and CD45⁺CD11b⁺ cells are shown perpendicularly. (*n* = 3 mice per group). PBMC: peripheral blood mononuclear cell. **b-d** Changes in the cell number and percentages (in CD45⁺ cells) of different IL-17A⁺ (**b**), IL-6⁺ (**c**), and RANKL⁺ (**d**) lineages in PBMC are shown perpendicularly (*n* = 3 mice per group). Data are presented as the mean ± SEM. *P < 0.05; **P < 0.01; ***P < 0.001; ****P < 0.0001; ns (not significant), P > 0.05; by two-side unpaired t-test with Welch's correction. The exact P values are shown in Supplementary Data. 2. The related gating strategy was shown in Supplementary Fig.29. Source data are provided as a Source Data file.

Supplementary Fig. 12

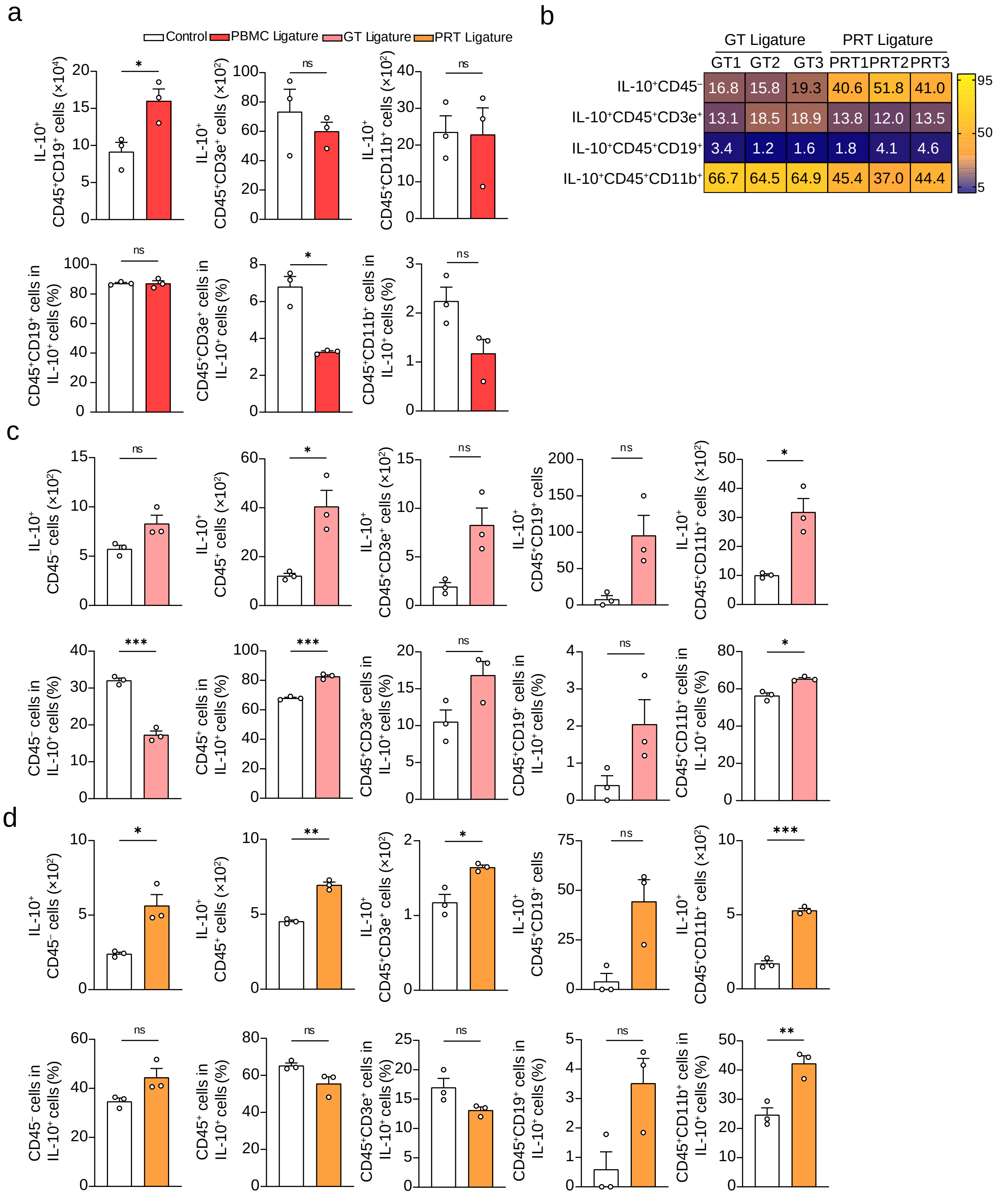


**Supplementary Fig. 12 Source of IL-10 on Day 5. | a** Changes in the cell number and percentages (in IL-10$^{+}$CD45$^{+}$ cells) of the different IL-10$^{+}$ lineages in PBMC are shown perpendicularly (*n* = 3 mice per group). PBMC: peripheral blood mononuclear cell. **b** Heat map of the percentage of different IL-10$^{+}$ lineages in the ligatured GT and PRT. The data from three independent experiments are shown (*n* = 3 mice per group, GT/PRT 1, 2, 3). GT: gingiva tissue, PRT: peri-root tissue **c, d** Changes in the cell number and percentages (in IL-10$^{+}$ or IL-10$^{+}$CD45$^{+}$ cells) of different IL-10$^{+}$ lineages in GT (**c**) and PRT (**d**) are shown perpendicularly (*n* = 3 mice per group). Data are presented as the mean ± SEM. *P < 0.05; **P < 0.01; ***P < 0.001; ns (not sgnificant), P > 0.05; by two-side unpaired t-test with Welch's correction. The exact P values are shown in Supplementary Data. 2. The related gating strategy was shown in Supplementary Fig.29. Source data are provided as a Source Data file.

Supplementary Fig.13

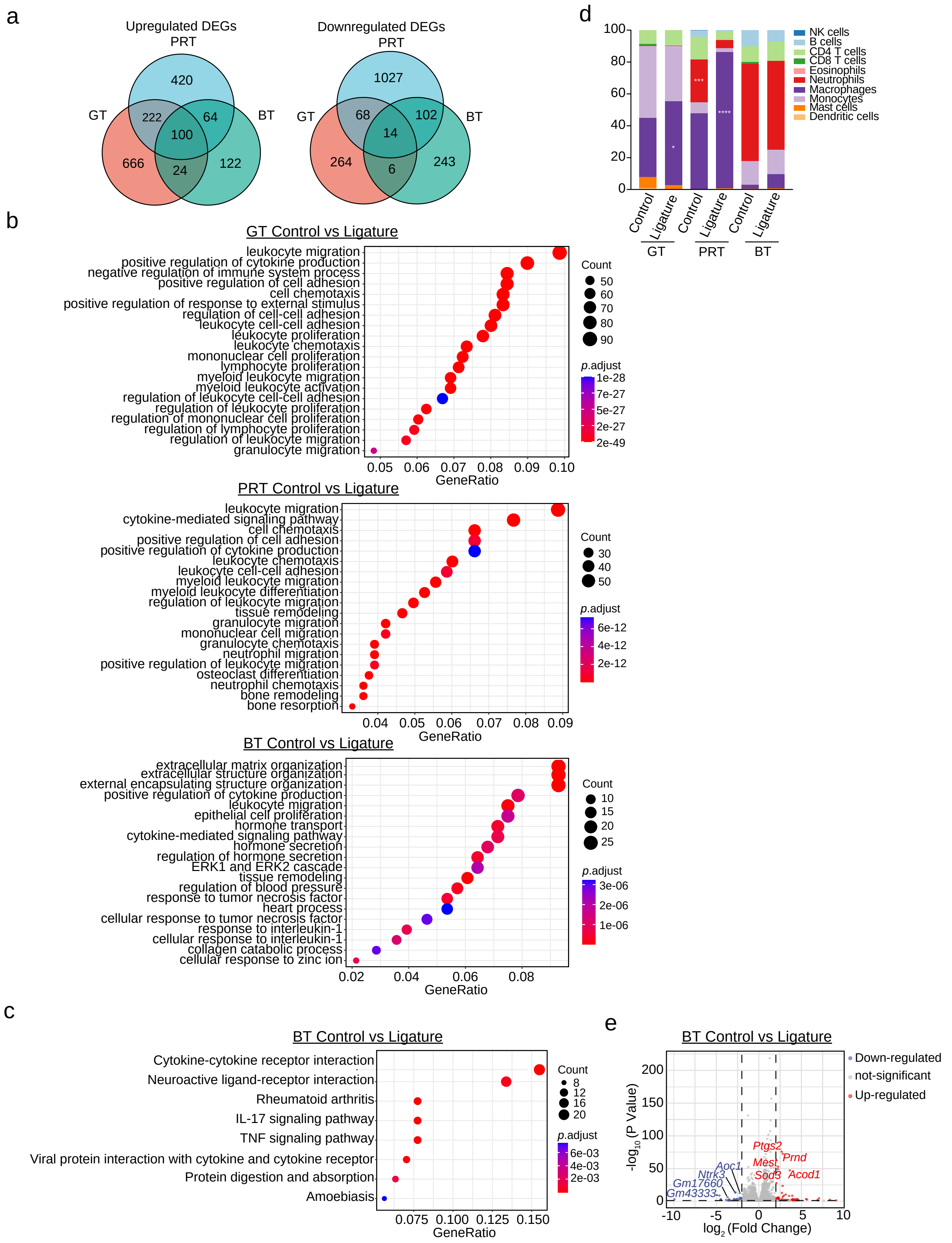

**Supplementary Fig. 13 Comprehensive analysis of the three tissues on Day 5. | a** Venn diagrams of differentially expressed genes (DEG) in three tissues. GT: gingiva tissue, PRT: peri-root tissue, BT: bone tissue. **b** GO term (BP) enrichment analysis in the three tissues by the upregulated DEGs using GO/KEGG enrichment analysis tools based on clusterplofiler in Hiplot (Benjamini & Hochbergmethod-adjusted P value < 0.01; false discovery rate < 0.05; fold-change of normalized counts ≥ 2). The top 20 terms are presented with their expression counts and adjusted (Benjamini & Hochberg method) P value. **c** KEGG pathway enrichment analysis by the upregulated DEGs in BT using GO/KEGG enrichment analysis tools based on clusterplofiler in Hiplot (Benjamini & Hochbergmethod-adjusted P value < 0.05; false discovery rate < 0.1; fold-change of normalized counts ≥ 2). The top 20 terms are presented with their expression counts and adjusted (Benjamini & Hochberg method) P value. **d** Immune cell component prediction by ImmuCC algorism. The population percentage was compared between the control and ligature samples within each tissue. *P < 0.05; ***P < 0.001; ****P < 0.0001; ns (not significant), P > 0.05; by two-way ANOVA with multiple comparisons via Tukey's test. The exact P values are shown in Supplementary Data. 2. Source data are provided as a Source Data file. **e** Volcano plots of the DEGs in BT. The top 5 genes with the highest adjusted P value are annotated.

Supplementary Fig.14

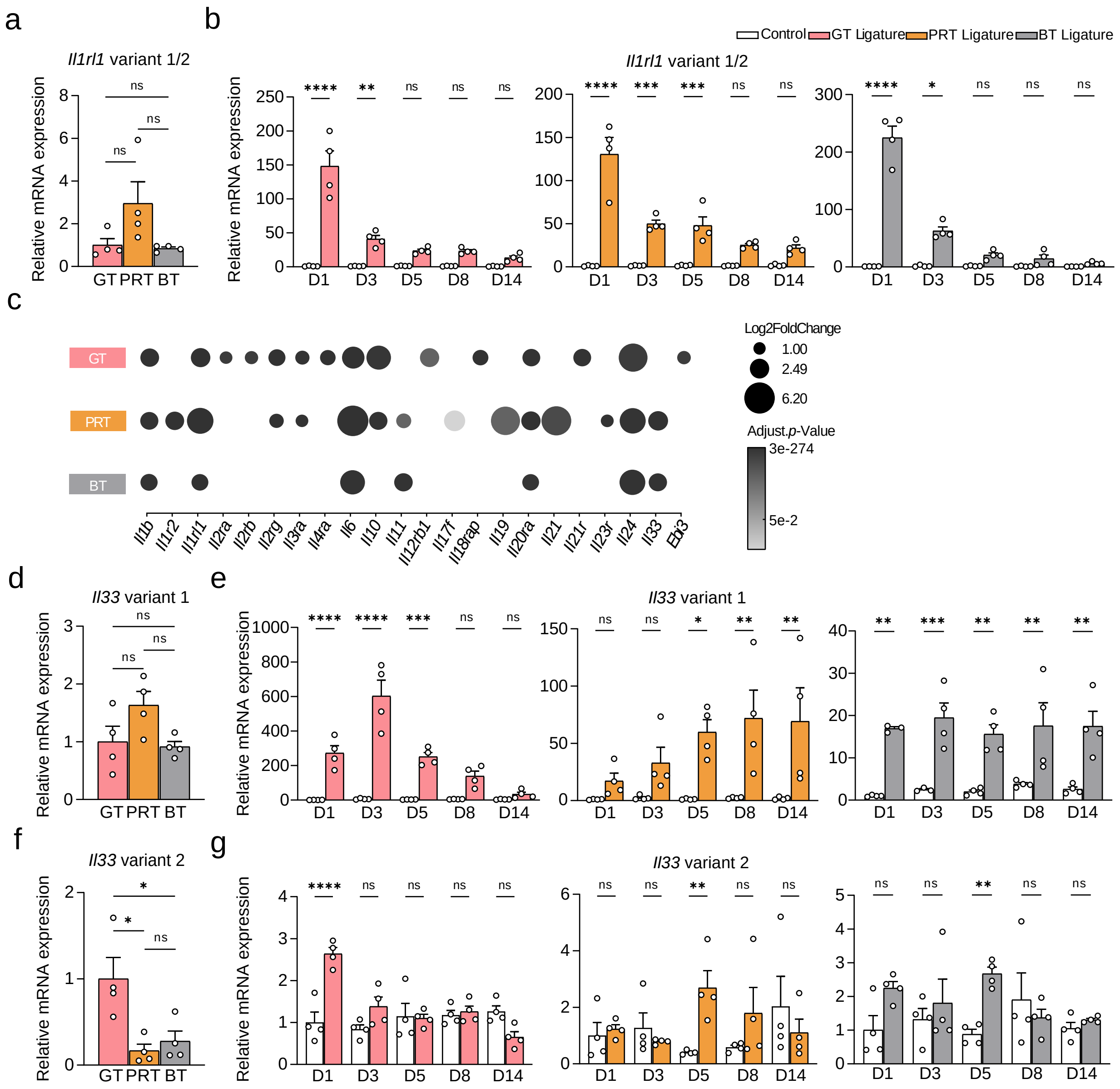

**Supplementary Fig. 14 Involvement of different transcriptional variants of the IL-33/ST2 axis. | a, d, f** mRNA transcriptional variant expression of *Il1rl1* variant 1/2 (**a**), *Il33* variant 1 (**d**), and *Il33* variant 2 (**f**) on the control side of the three tissues (GT = 1; *n* = 4 mice per group except for the group highlighted in Source Data file, which were *n* = 3 mice). GT: gingiva tissue, PRT: peri-root tissue, BT: bone tissue. **b, e, g** Temporal change in mRNA transcriptional variant expression of *Il1rl1* variant 1/2 (**b**), *Il33* variant 1 (**e**), and *Il33* variant 2 (**g**) in the three tissues (Control Day 1 of each tissue = 1; *n* = 4 mice per group except for the group highlighted in Source Data file, which were *n* = 3 mice). **c** Log2 fold change of interleukin-related mRNA in the RNA-seq data of the three tissues. The fold changes calculated from the raw expression counts via DEseq2 are presented with their adjusted (Benjamini & Hochberg method) P value. The data for fold changes fewer than 1 or adjusted P -value over 0.1 are not shown. Data are presented as the mean ± SEM. *P < 0.05; **P < 0.01; ***P < 0.001; ****P < 0.0001; ns (not significant), P > 0.05; by one-way ANOVA with multiple comparisons via Tukey's test (**a, d, f**); and two-way ANOVA with multiple comparisons via Šídák's method (**b, e, g**). The obvious outliers were evaluated and excluded by Grubb's test (α = 0.05). The exact P values are shown in Supplementary Data. 2. Source data are provided as a Source Data file.

Supplementary Fig. 15

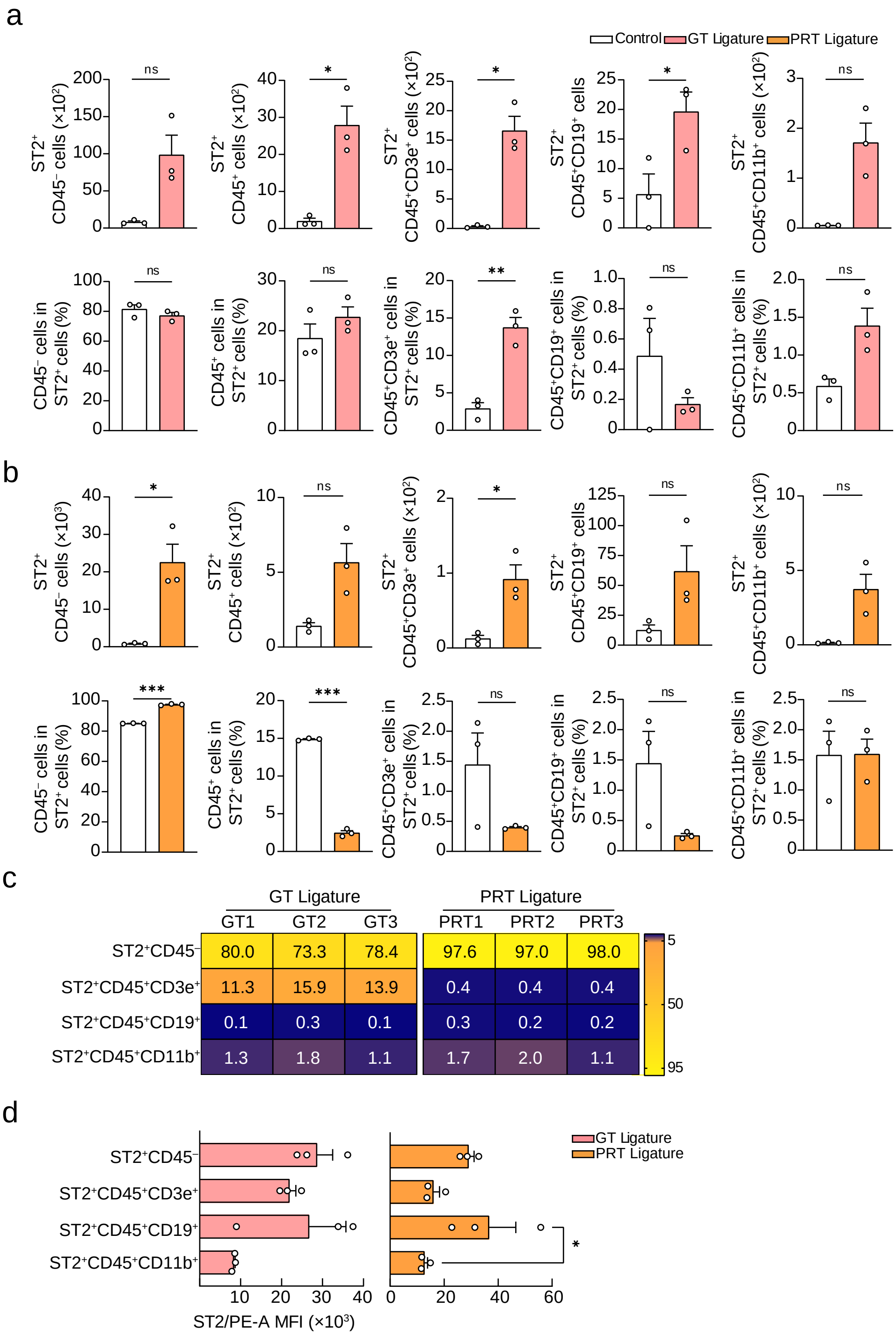


**Supplementary Fig. 15 ST2 positive cells in GT and PRT in on Day 5. | a, b** Changes in the cell number and percentages (in ST2$^+$ or ST2$^+$CD45$^+$ cells) of the different ST2$^+$ lineages in GT (**a**) and PRT (**b**) are shown perpendicularly (*n* = 3 mice per group). GT: gingiva tissue, PRT: peri-root tissue. **c** Heat map of the percentage of different ST2$^+$ lineages in ligated GT and PRT. The data from three independent experiments are shown (*n* = 3 mice per group, GT/PRT 1, 2, 3). **d** MFI (median fluorescence intensity) of different levels of ST2$^+$ in ligated GT and PRT (*n* = 3 mice per group). Data are presented as the mean ± SEM. *P < 0.05; **P < 0.01; ***P < 0.001; ns (not significant), P > 0.05; by two-side unpaired t-test with Welch's correction (**a, b**); by one-way ANOVA with multiple comparisons via Tukey's test (**d**). The exact P values are shown in Supplementary Data. 2. The related gating strategy was shown in Supplementary Fig. 29. Source data are provided as a Source Data file.

Supplementary Fig. 16

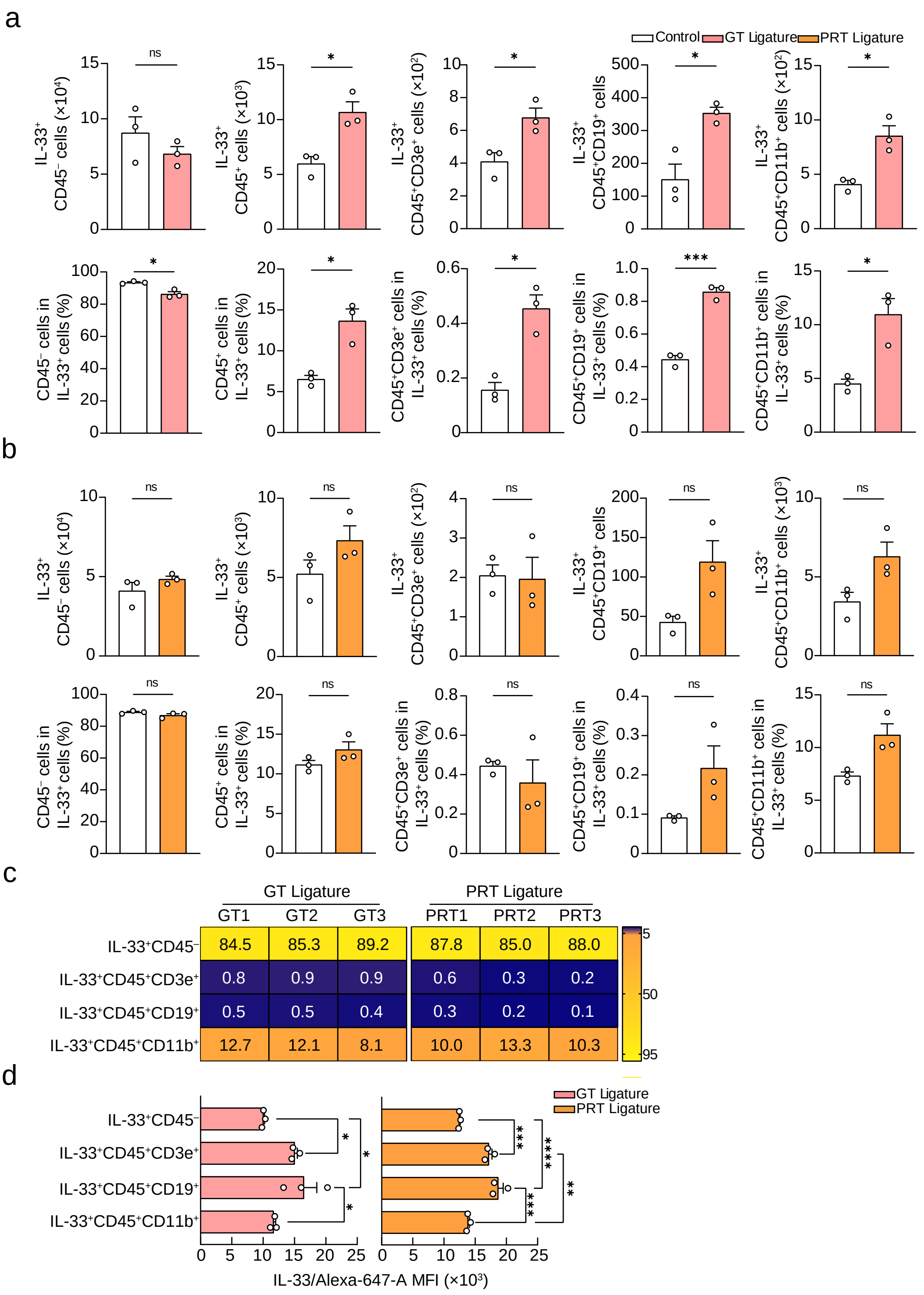


**Supplementary Fig. 16 Source of IL-33 in GT and PRT on Day 5. | a, b** Changes in the cell number and percentages (in IL-33$^+$ or IL-33$^+$CD45$^+$ cells) of the different IL-33$^+$ lineages in GT (**a**) and PRT (**b**) are shown perpendicularly (*n* = 3 mice per group). GT: gingiva tissue, PRT: peri-root tissue. **c** Heat map of the percentage of different IL-33$^+$ lineages in ligated GT and PRT. The data from three independent experiments are shown (*n* = 3 mice per group, GT/PRT 1, 2, 3). **d** MFI (median fluorescence intensity) of different levels of IL-33$^+$ cells in ligated GT and PRT (*n* = 3 mice per group). Data are presented as the mean ± SEM. *P < 0.05; **P < 0.01; ***P < 0.001; ****P < 0.0001; ns (not significant), P > 0.05; by two-side unpaired t-test with Welch's correction; by one-way ANOVA with multiple comparisons via Tukey's test (**d**). The exact P values are shown in Supplementary Data. 2. The related gating strategy was shown in Supplementary Fig. 29. Source data are provided as a Source Data file.

Supplementary Fig. 17

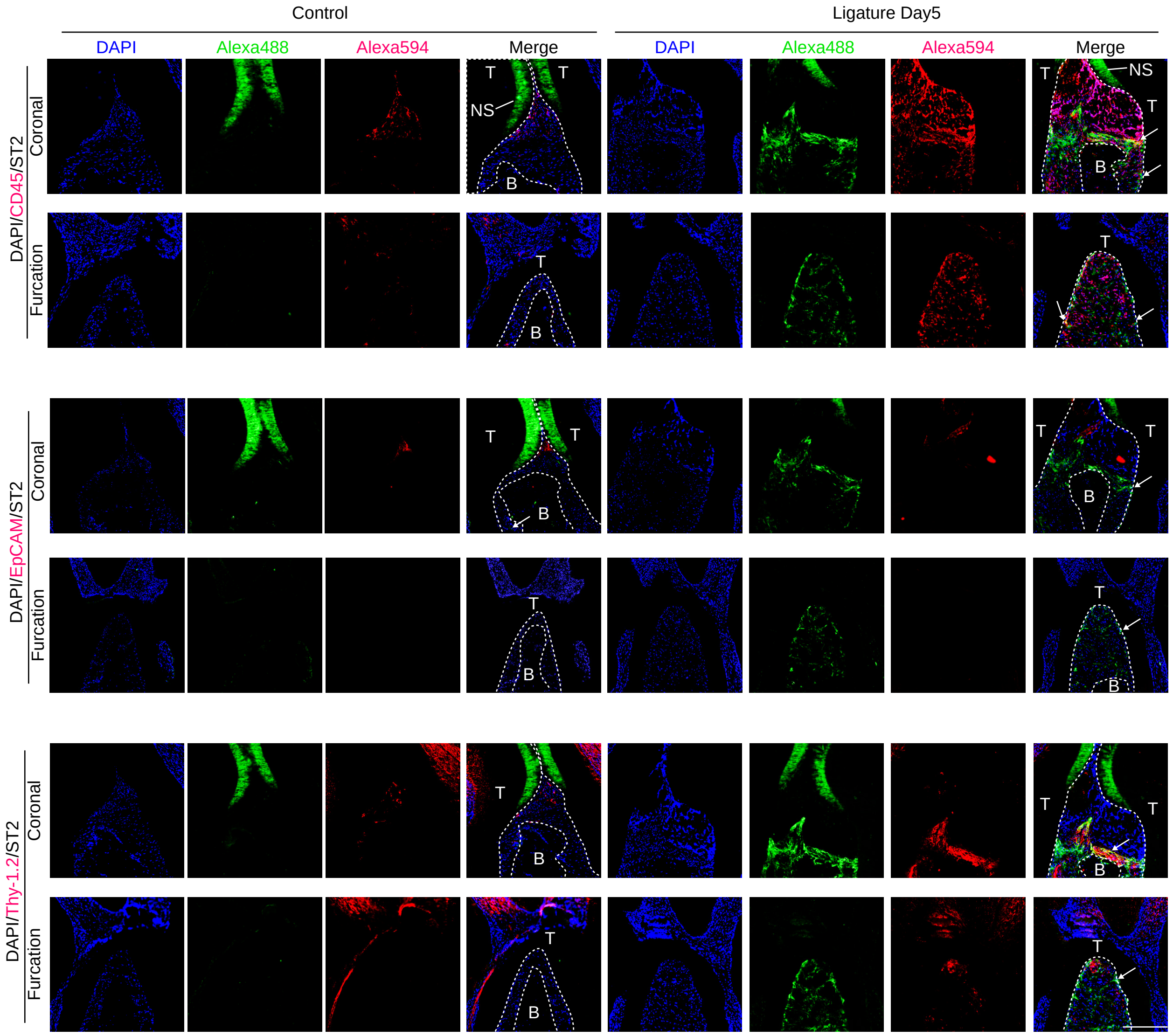


**Supplementary Fig. 17 Immunofluorescence staining of ST2 on Day 5 with higher resolution.** | Representative immunofluorescence staining images from three independent mouse experiments of ST2 with membrane marker proteins in the second molar region. T, Tooth; NS, nonspecific signal; B, bone tissue. The white arrows indicate the different ST2 positive cells. Scale bar, 60 μm.

Supplementary Fig. 18

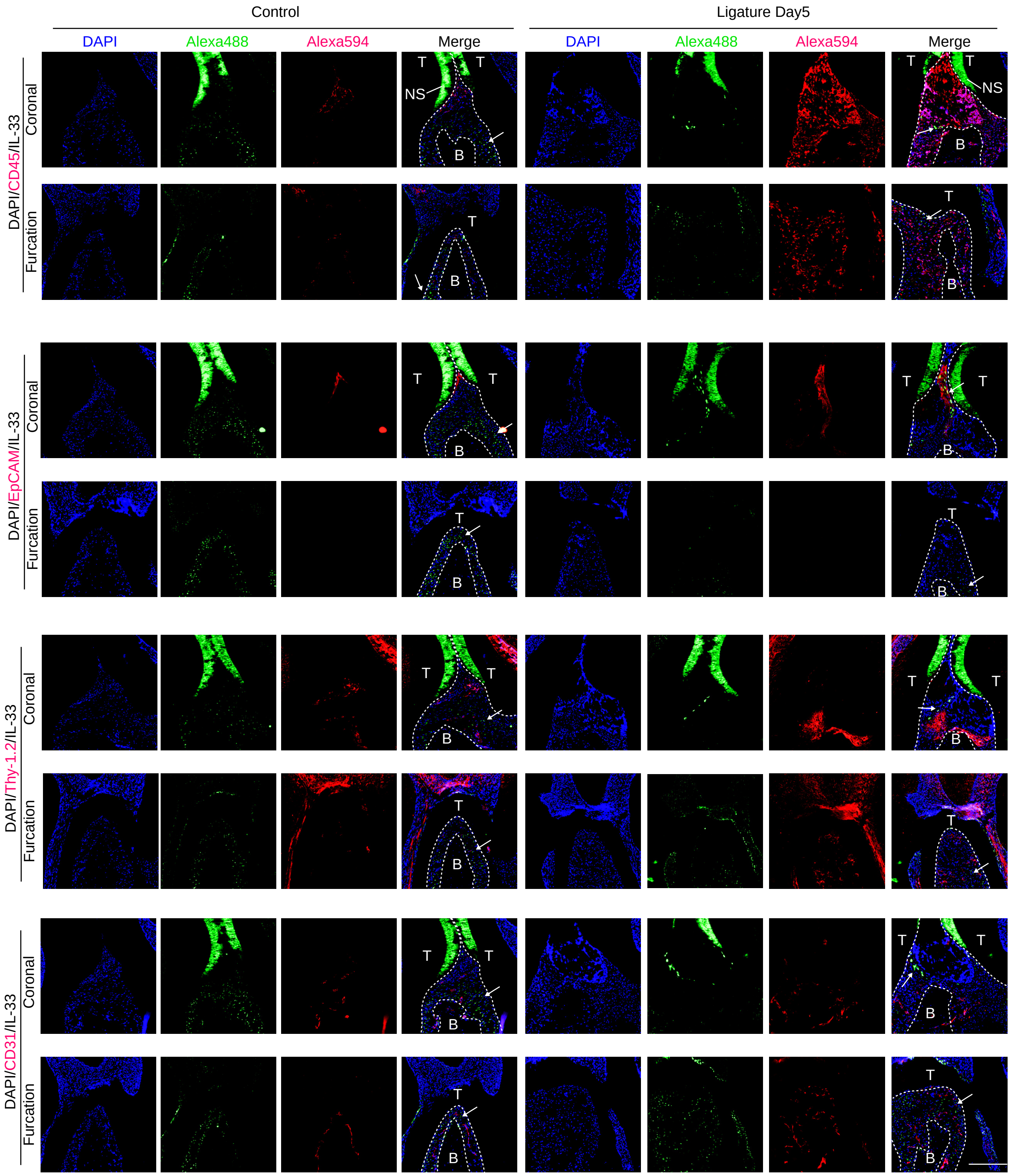


**Supplementary Fig. 18 Immunofluorescence staining of IL-33 on Day 5 with higher resolution.** | Representative immunofluorescence staining images from three independent mouse experiments of IL-33 with membrane marker proteins in the second molar region. T, Tooth; NS, nonspecific signal; B, bone tissue. The white arrows indicate the different IL-33 positive cells. Scale bar, 60 μm.

Supplementary Fig. 19

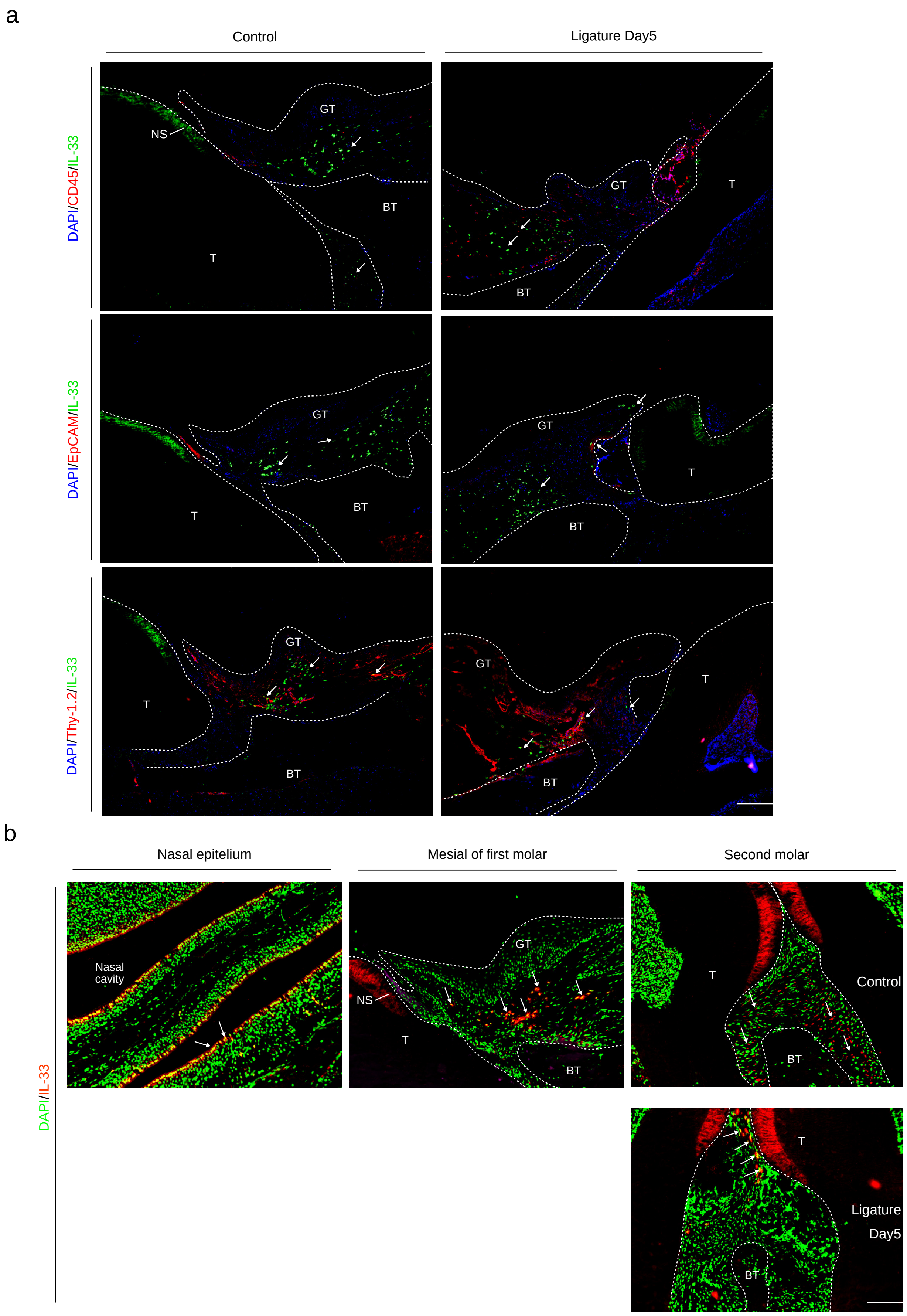

**Supplementary Fig. 19 Investigating the IL-33 high productive cells and the location of IL-33 within the cell. | a** Representative immunofluorescence staining images from three independent mouse experiments of IL-33 with membrane marker proteins in the mesial first molar region. The white arrows indicate the IL-33 positive cells in the mesial area of the first molar. Scale bar, 300 μm. **b** Representative immunofluorescence staining images from three independent mouse experimens of IL-33 showing different patterns of intracellular localization. T, Tooth; GT, gingival tissue; BT, bone tissue. The white arrows indicate the different IL-33 positive cells. Scale bar, 60 μm.

Supplementary Fig. 20

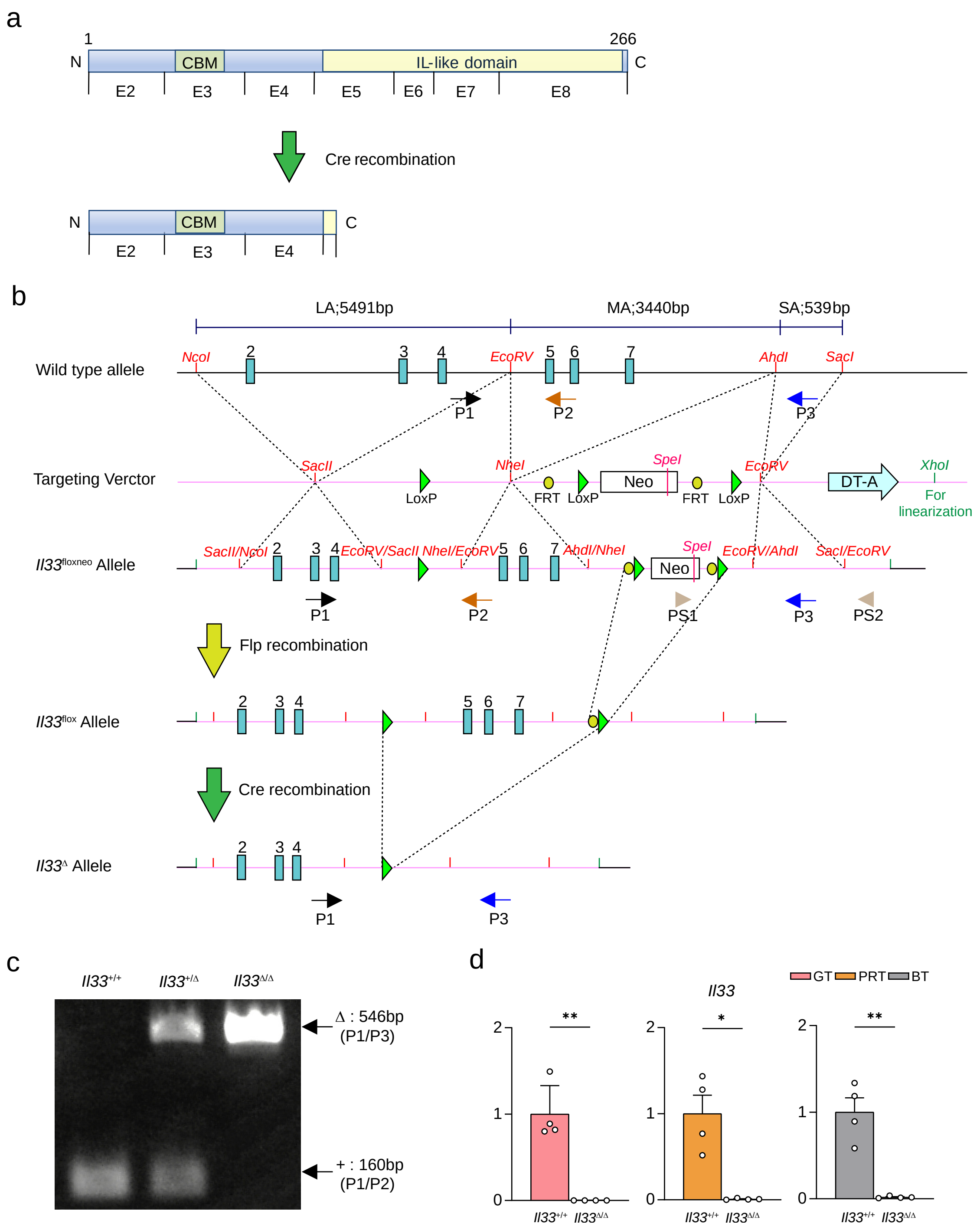

**Supplementary Fig. 20 Conditional gene-targeting of *Il33*. | a** Representation of the functional extracellular IL-like domain of IL-33 binding to its receptor. Cre-mediated excision of exons 5 to 7 of the *Il33* gene led to a loss of the IL-like domain of IL-33. CBM: chromatin binding motif. E: exon. **b** Targeting strategy to generate *Il33* conditional mutant mice. The genomic structure of the wild-type *Il33* gene, the targeting vector and the targeted alleles are indicated. Exons 5 to 7 are flanked by *loxP* sequences; the *Neo* cassette is flanked by *frt* sequences. The modified *Il33* locus after homologous recombination ($Il33^{floxNeo}$ allele), the *Il33* gene after excision of the *Neo* cassette following expression of Flp recombinase ($Il33^{flox}$ allele), and the deleted *Il33* gene after Cre-mediated excision of the exons ($Il33^{\Delta}$ allele) are shown. The arrows below the diagram of the wild-type allele indicate the positions of the primers (P1, P2 and P3) used for PCR genotyping. **c** Representive image of genotyping PCR agarose gel electrophoresis of tail genomic DNA using the primer pairs shown in Supplementary Text 1. **d** mRNA expression of *Il33* on the control side of the different periodontal tissues between the WT, $Il33^{+/\Delta}$ and $Il33^{\Delta/\Delta}$ strains (on Day 5; each periodontal tissue of wild-type strain = 1; $n$ = 4 mice per group). GT: gingiva tissue, PRT: peri-root tissue, BT: bone tissue. Data are presented as the mean ± SEM. *$P < 0.05$; **$P < 0.01$; by Welch's t-test (**d**). The exact P values are shown in Supplementary Data. 2. Source data are provided as a Source Data file.

Supplementary Fig. 21

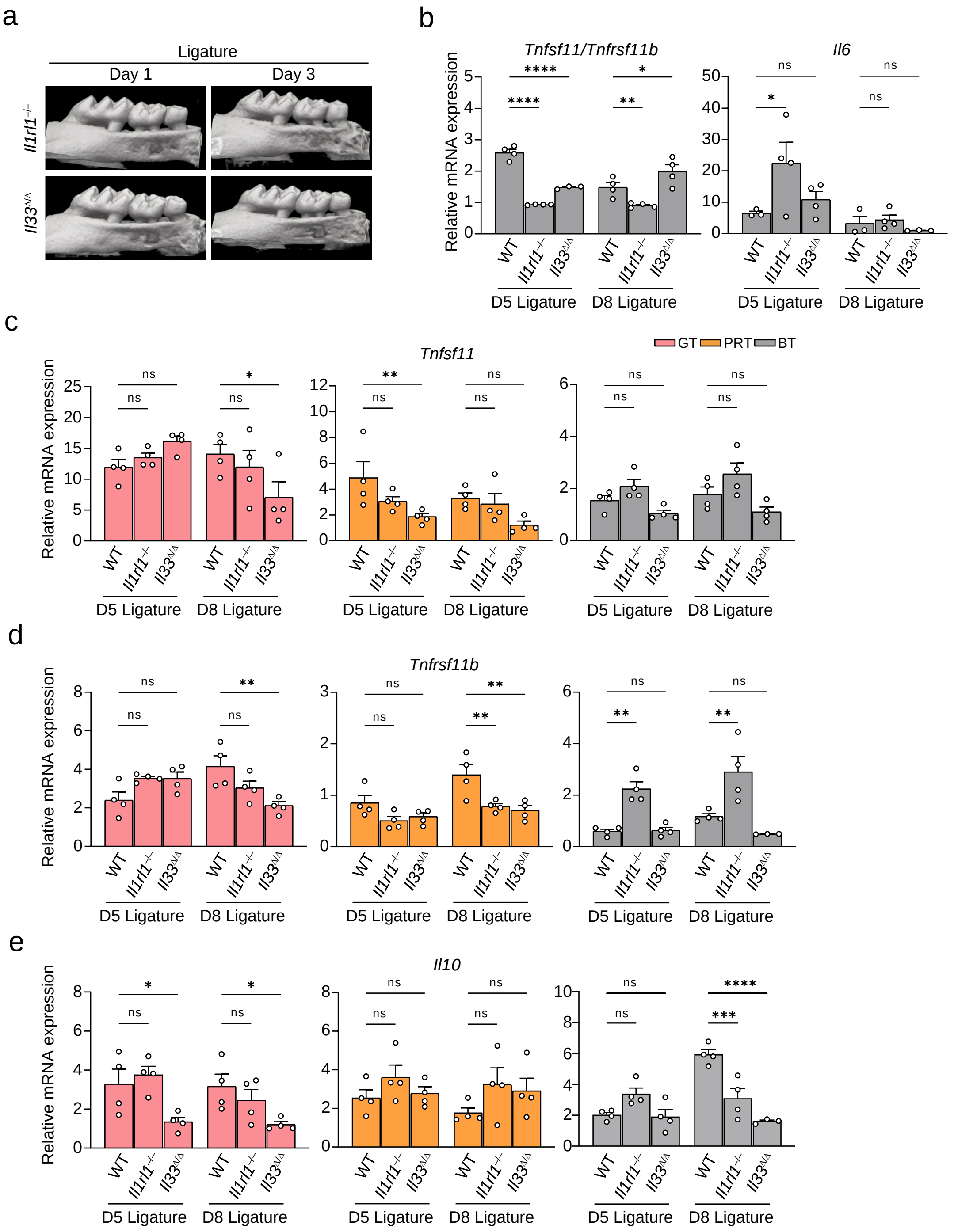


**Supplementary Fig. 21 Additional information for analyzing the mechanism of the protective role of the IL-33/ST2 axis. | a** Representative maxillary μCT images of KO mice on the ligature sides on Day 1 and Day 3 ($n$ = 4 mice per group). Scale bar, 1 mm. **b** Temporal changes in the *Tnfsf11*/*Tnfrsf11b* mRNA ratio and mRNA expression of *Il6* in BT among the mouse strains (control Day 5 of each tissue = 1; $n$ = 4 mice per group except for the group highlighted in Source Data file, which were $n$ = 3 mice). BT: bone tissue.**c-e** Temporal changes in *Tnfsf11* (**c**), *Tnfrsf11b* (**d**), and *Il10* (**e**) in the three tissues among the mouse strains (control Day 5 of each tissue in each strain = 1; $n$ = 4 mice per group except for the group highlighted in Source Data file, which were $n$ = 3 mice). D5: Day 5; D8: Day 8. GT: gingiva tissue, PRT: peri-root tissue. Data are presented as the mean ± SEM. *P < 0.05; **P < 0.01; ***P < 0.001; ****P < 0.0001; ns (not sgnificant), P > 0.05; by two-way ANOVA with multiple comparisons via Dunnett's test. The obvious outliers were evaluated and excluded by Grubb's test ($\alpha$ = 0.05). The exact P values are shown in Supplementary Data. 2. Source data are provided as a Source Data file.

Supplementary Fig. 22

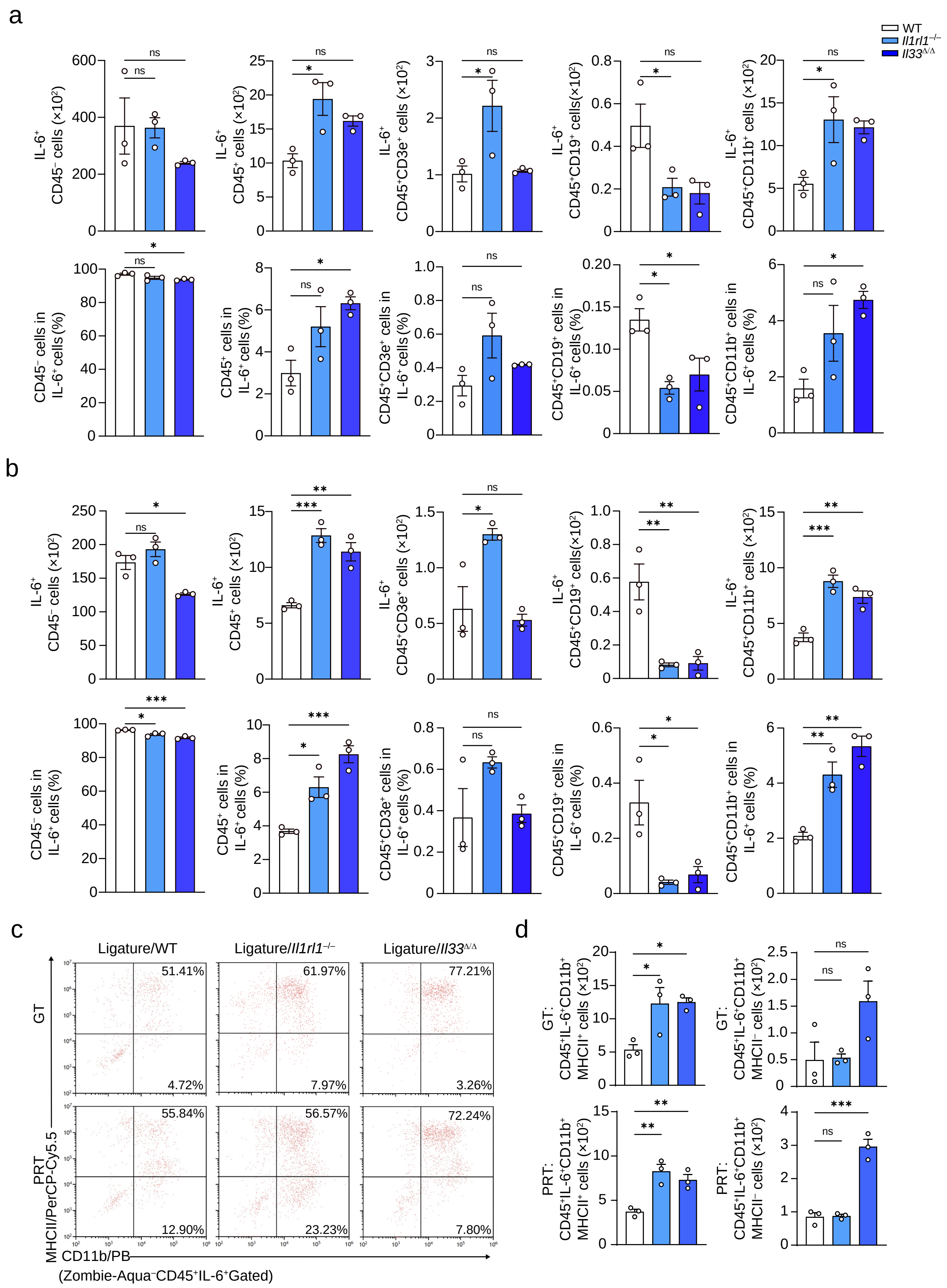

**Supplementary Fig. 22 IL-6 positive cell component alteration among the three mouse strains on Day 5. | a, b** Changes in the cell number and percentages (in IL-$6^+$ or IL-$6^+$CD$45^+$ cells) of different IL-$6^+$ lineages in GT (**a**) and PRT (**b**) are shown perpendicularly (*n* = 3 mice per group). GT: gingiva tissue, PRT: peri-root tissue. **c** Changes in IL-$6^+$ cells in GT and PRT with ligature placement between three mouse strains. A plot merged the data of three individual mouse experiments is shown, and the percentages presented in the gates are the mean of them. **d** Changes in the cell number of IL-$6^+$CD$45^+$CD11$b^+$MHCII$^+$ or IL-$6^+$CD$45^+$CD11$b^+$MHCII$^-$ lineages in GT and PRT are shown perpendicularly (*n* = 3 mice per group). Data are presented as the mean ± SEM. *P < 0.05; **P < 0.01; ***P < 0.001; ns (not significant), P > 0.05; by one-way ANOVA with multiple comparisons via Dunnett's test. The exact P values are shown in Supplementary Data. 2. The related gating strategy was shown in Supplementary Fig. 29. Source data are provided as a Source Data file.

Supplementary Fig. 23

a

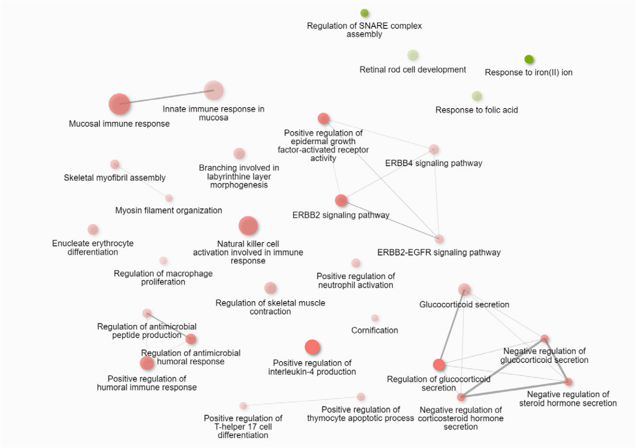


b

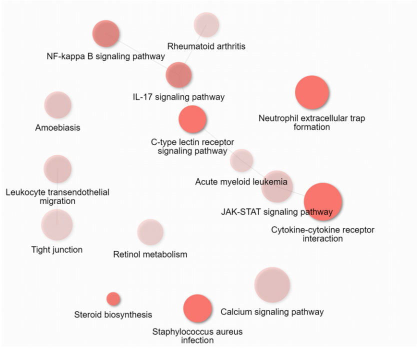


c

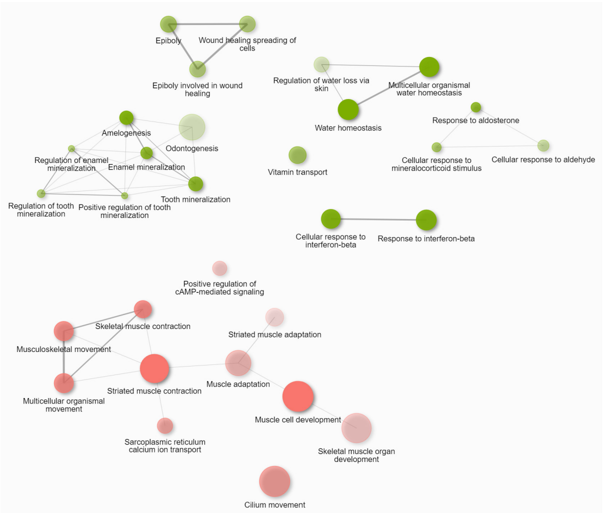


d

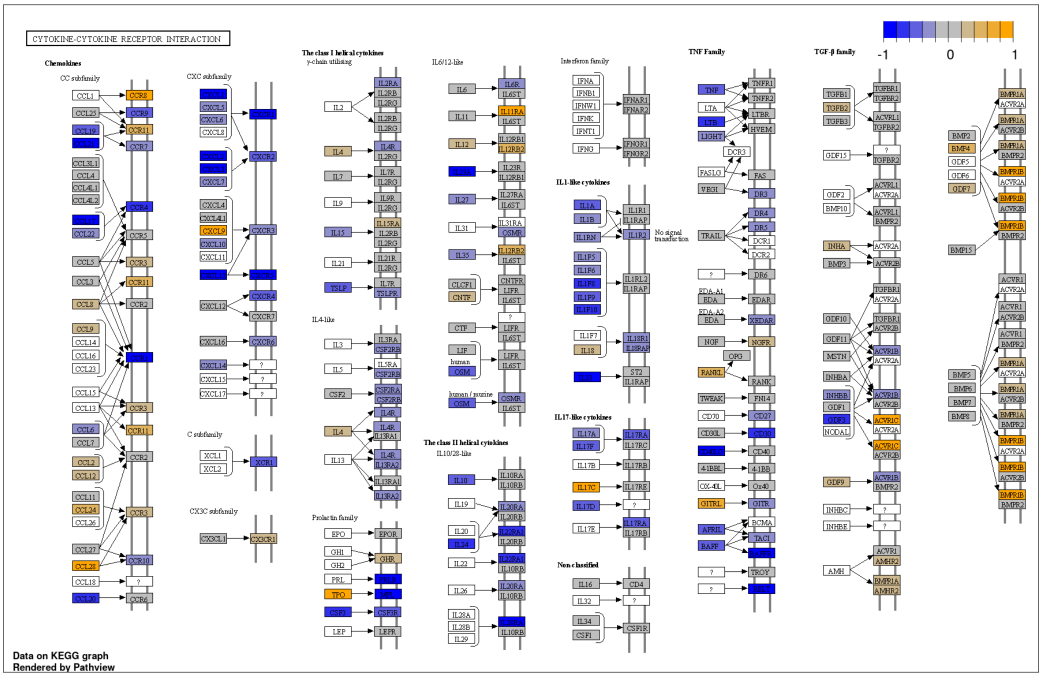

**Supplementary Fig. 23 Comprehensive analysis of periodontitis-induced BT and PRT between WT and *Il33*-deficient mice on Day 11. | a, c** Pathway analysis of GO term (BP) in PRT (**a**) and BT (**c**) by iDEP pipeleine. Green bubbles repre-sent upregulated pathways, and the red bubbles represent downregulated path-ways. The top 30 terms are presented, and the details are in Supplementary Table 1 and 3. PRT: peri-root tissue, BT: bone tissue. **b** Pathway analysis of KEGG pathways in PRT by iDEP pipeleine. Red bubbles represent downregulated pathways. The top 30 pathways are presented, and the details are in the Supplementary Table. 2. **d** Specific enriched genes in the PRT's "Cytokine-Cy-tokine receptor interaction" KEGG pathway of the PRT by iDEP pipeleine. Yellow represents upregulated, and blue represents downregulated.

Supplementary Fig. 24

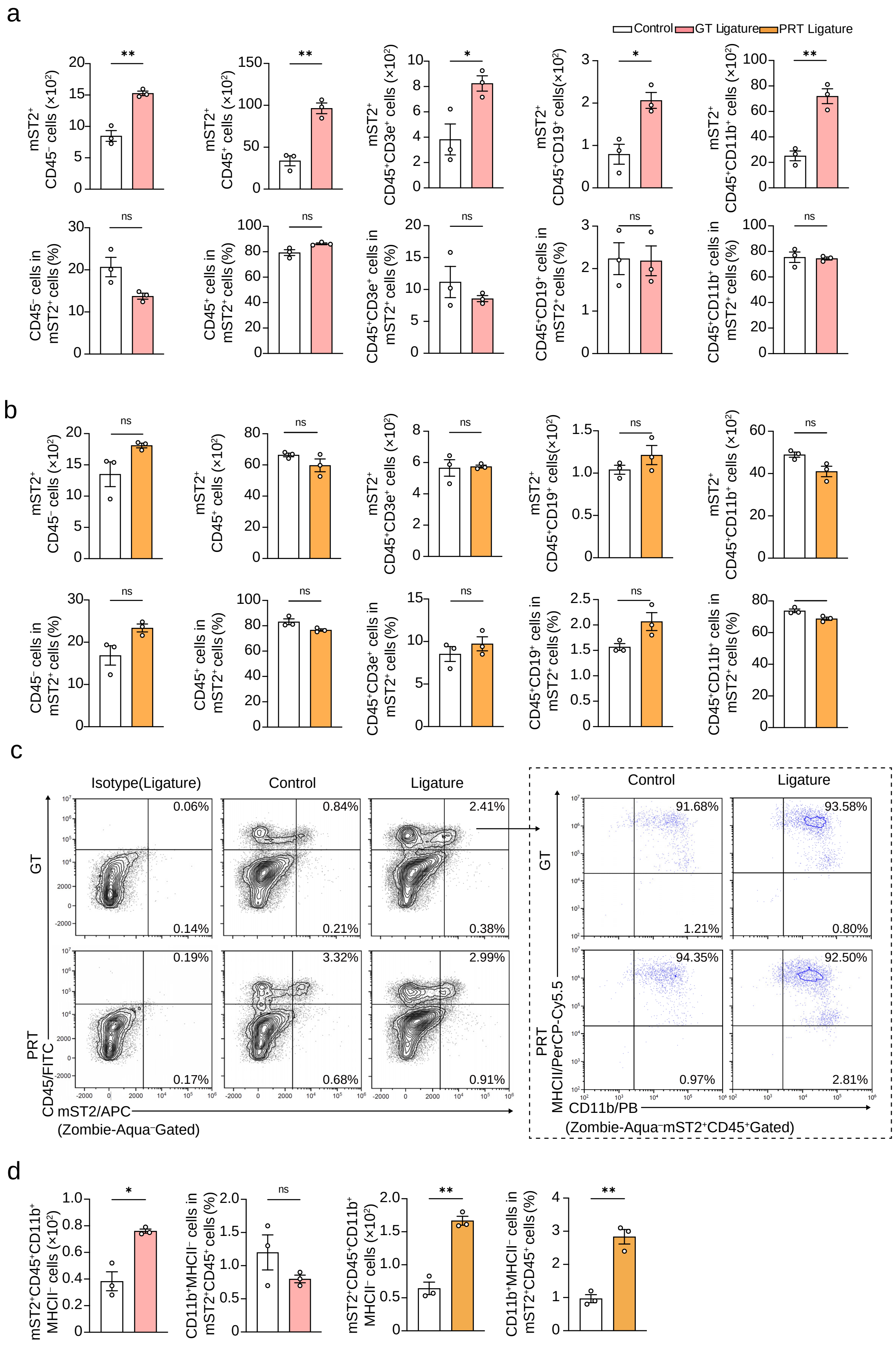

**Supplementary Fig. 24 mST2 positive cell component alteration in the GT and PRT on Day 5. | a, b** Changes in the cell number and percentages (in mST2$^{+}$ or mST2$^{+}$CD45$^{+}$ cells) of different mST2$^{+}$ lineages in GT (**a**) and PRT (**b**) are shown perpendicularly (*n* = 3 mice per group). GT: gingiva tissue, PRT: peri-root tissue, BT: bone tissue. **c** Changes in mST2$^{+}$CD45$^{+}$ and mST2$^{+}$CD45$^{+}$CD11b$^{+}$MHCII$^{+}$, and mST2$^{+}$CD45$^{+}$CD11b$^{+}$MHCII$^{-}$ cells in GT and PRT with or without ligature placement. A representative contour plot is shown, and the percentages shown in the gates are the mean of three individual mice experiments. **d** Changes in the cell number and percentages (in mST2$^{+}$CD45$^{+}$ cells) of mST2$^{+}$CD45$^{+}$CD11b$^{+}$MHCII$^{-}$ lineages in GT and PRT are shown perpendicularly (*n* = 3 mice per group). Data are presented as the mean ± SEM. *P < 0.05; **P < 0.01; ns, P > 0.05; by two-side unpaired t-test with Welch's correction. The exact P values are shown in Supplementary Data. 2. The related gating strategy was shown in Supplementary Fig. 29. Source data are provided as a Source Data file.

Supplementary Fig. 25

a

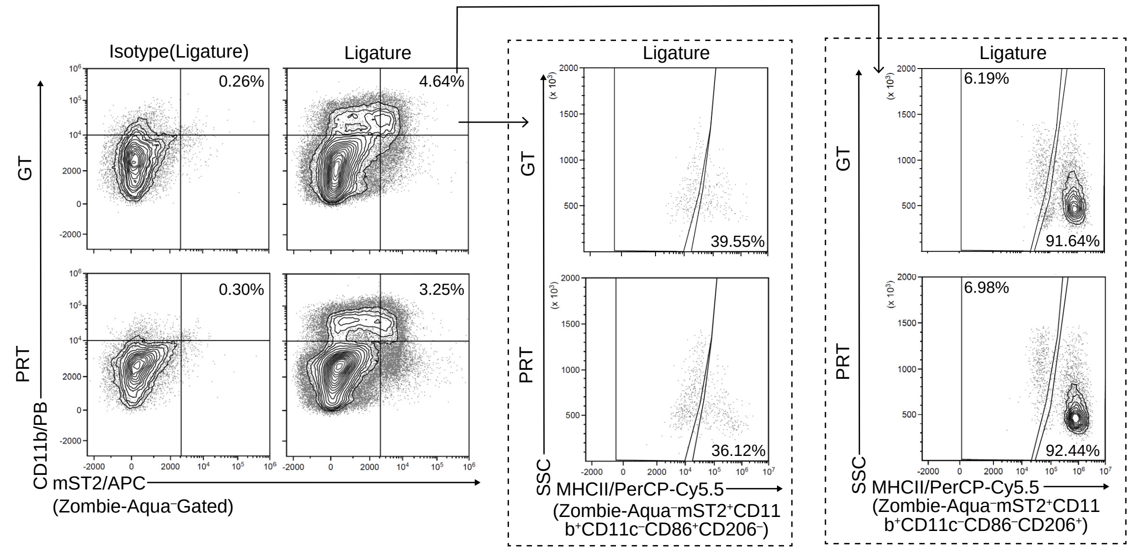


b

Percentages in CD11b⁺MHCII⁻mST2⁺ cells

| | GT Ligature | | | PRT Ligature | | |
|---|---|---|---|---|---|---|
| | GT1 | GT2 | GT3 | PRT1 | PRT2 | PRT3 |
| mST2⁺CD11b⁺MHCII⁻CD115⁺ | 12.0 | 9.9 | 10.8 | 5.6 | 7.0 | 4.3 |
| mST2⁺CD11b⁺MHCII⁻CD115⁻Ly-6G⁺ | 4.2 | 3.4 | 5.4 | 6.0 | 4.9 | 2.8 |
| mST2⁺CD11b⁺MHCII⁻CD115⁻Ly-6G⁻c-kit⁺ | 77.3 | 80.8 | 77.6 | 77.1 | 78.5 | 82.4 |

Color scale: 5, 50, 95

c

Percentages in each lineage

| | GT Ligature | | | PRT Ligature | | |
|---|---|---|---|---|---|---|
| | GT1 | GT2 | GT3 | PRT1 | PRT2 | PRT3 |
| mST2⁺CD11b⁺MHCII⁻CD115⁺ | 43.4 | 24.1 | 26.9 | 20.9 | 18.8 | 13.7 |
| mST2⁺CD11b⁺MHCII⁻CD115⁻Ly-6G⁺ | 1.5 | 0.8 | 1.3 | 1.2 | 0.9 | 0.7 |
| mST2⁺CD11b⁺MHCII⁻CD115⁻Ly-6G⁻c-kit⁺ | 22.0 | 23.0 | 18.0 | 15.1 | 11.3 | 13.1 |

Color scale: 5, 50, 95

d

e

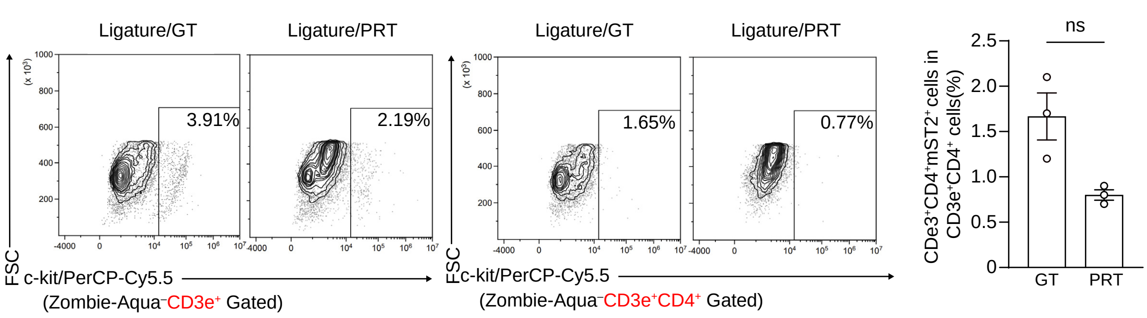

**Supplementary Fig. 25 mST2-positive myeloid cell component alteration in the GT and PRT on Day 5. | a** Changes in $mST2^+CD11b^+$, $mST2^+CD11b^+CD11c^-MHCII^+CD86^+CD206^-$, $mST2^+CD11b^+CD11c^-MHCII^+CD86^-CD206^+$, and $mST2^+CD11b^+CD11c^-MHCII^-CD86^-CD206^+$ cells in GT and PRT. A plot merged the data of three individual mouse experiments is shown, and the percentages presented in the gates are the mean of them. GT: gingiva tissue, PRT: peri-root tissue. **b, c** Heat map of the percentage of different $mST2^+CD11b^+MHCII^-$ lineages in $mST2^+CD11b^+$ cells (**b**), and cells of each lineage type (**c**). $n = 3$ mice per group. **d** The percentage of $mST2^+CD3\varepsilon^+$ and $mST2^+CD3\varepsilon^+CD4^+$ cells in GT and PRT. A plot merged the data of three individual mouse experiments is shown, and the percentages presented in the gates are the mean of them. **e** Changes in the cell percentages (in $CD3e^+CD4^+$ cells) of $mST2^+CD3e^+CD4^+$ in GT and PRT ($n = 3$ mice per group). Data are presented as the mean ± SEM. ns (not significant), $P > 0.05$; by two-side unpaired t-test with Welch's correction. The exact P values are shown in Supplementary Data. 2. The related gating strategy was shown in Supplementary Fig. 31 (**a**), 32 (**b, c**), 33a (**d, e**). Source data are provided as a Source Data file.

Supplementary Fig. 26

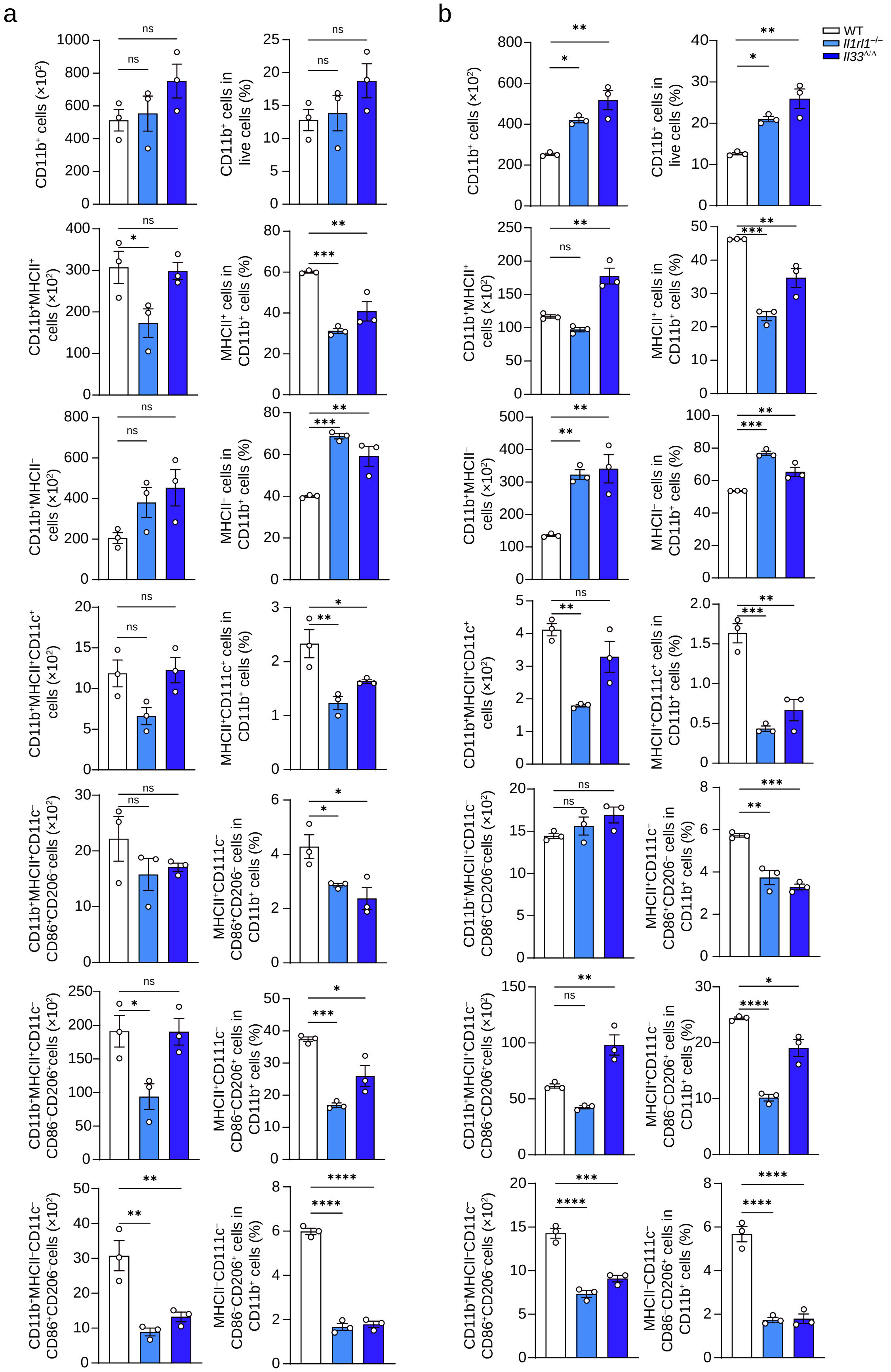

**Supplementary Fig. 26 Antigen-presenting myeloid cell component alteration among the three mouse strains on Day 5. | a, b** Changes in the cell number and percentages (in $CD11b^+$ cells) of different myeloid cell lineages in GT (**a**) and PRT (**b**) are shown horizontally (*n* = 3 mice per group). GT: gingiva tissue, PRT: peri-root tissue. Data are presented as the mean ± SEM. *P < 0.05; **P < 0.01; ***P < 0.001; ****P < 0.0001; ns (not significant), P > 0.05; by one-way ANOVA with multiple comparisons via Dunnett's test. The exact P values are shown in Supplementary Data. 2. The related gating strategy was shown in Supplementary Fig. 31. Source data are provided as a Source Data file.

Supplementary Fig. 27

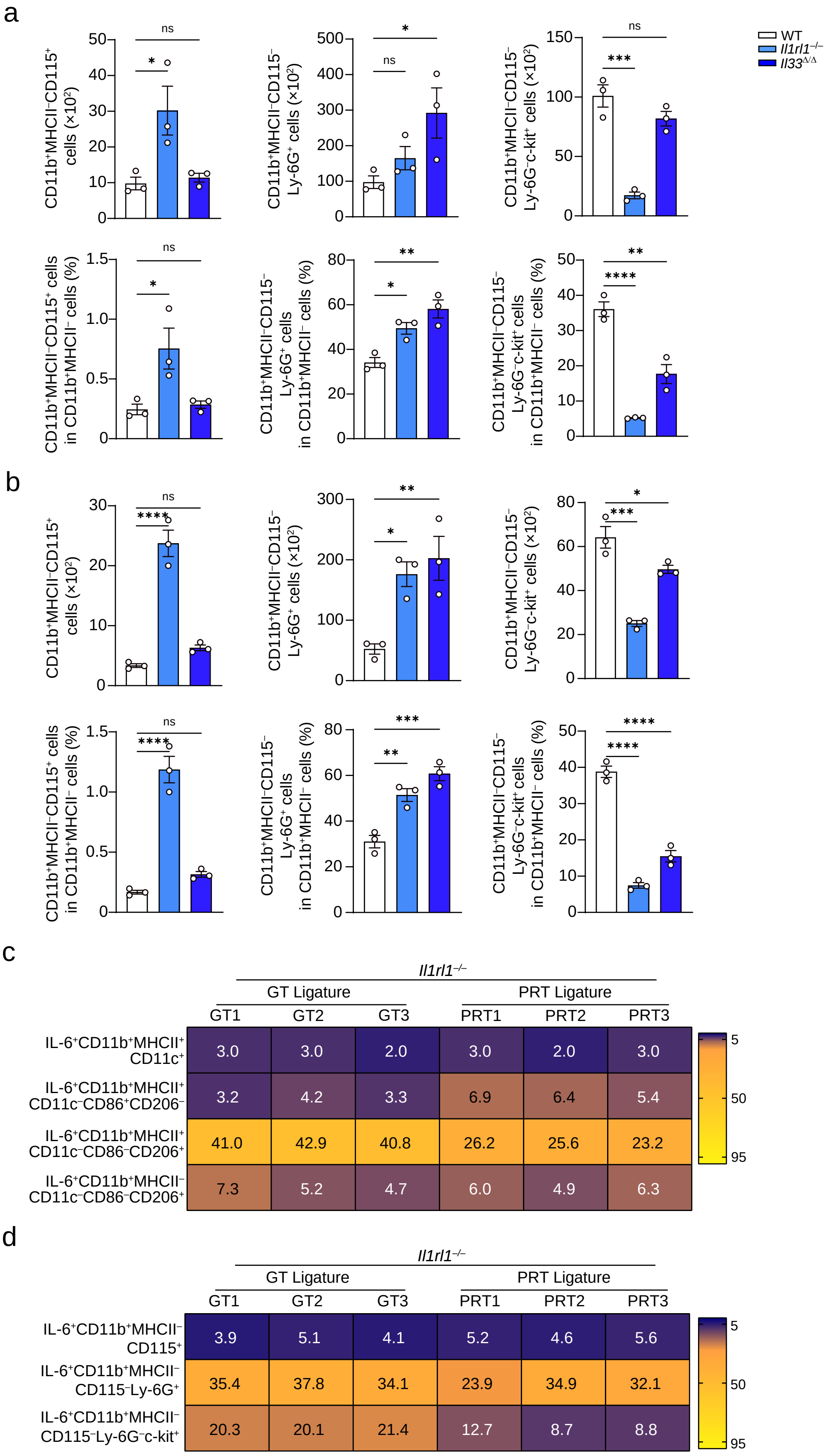

**Supplementary Fig. 27 Non-antigen-presenting myeloid cell component alteration among three mouse strains and IL-6$^+$ myeloid cells in GT and PRT of *Il1rl1*-deficient mice on Day 5. | a, b** Changes in the cell number and percentages (in CD11b$^+$MHCII$^-$ cells) of different myeloid cell lineages in GT (**a**) and PRT (**b**) are shown perpendicularly ($n$ = 3 mice per group). GT: gingiva tissue, PRT: peri-root tissue. **c** Heat map of the percentage of different mST2$^+$ lineages in ligated GT and PRT in *Il1rl1*$^{-/-}$ mice (in mST2$^+$CD11b$^+$ cells). The data from three independent experiments are shown ($n$ = 3 mice per group, GT/PRT 1, 2, 3). **d** Heat map of the positive percentage of different IL-6$^+$ non-antigen-presenting myeloid lineages in ligated GT and PRT (in IL-6$^+$CD11b$^+$MHCII$^-$ cells; $n$ = 3 mice per group, GT/PRT 1, 2, 3). Data are presented as the mean ± SEM. *$P < 0.05$; **$P < 0.01$; ***$P < 0.001$; ****$P < 0.0001$; ns (not significant), $P > 0.05$; by one-way ANOVA with multiple comparisons via Dunnett's test. The exact P values are shown in Supplementary Data. 2. The related gating strategy was shown in Supplementary Fig. 32 (**a, b, d**), 31 (**c**). Source data are provided as a Source Data file.

Supplementary Fig. 28

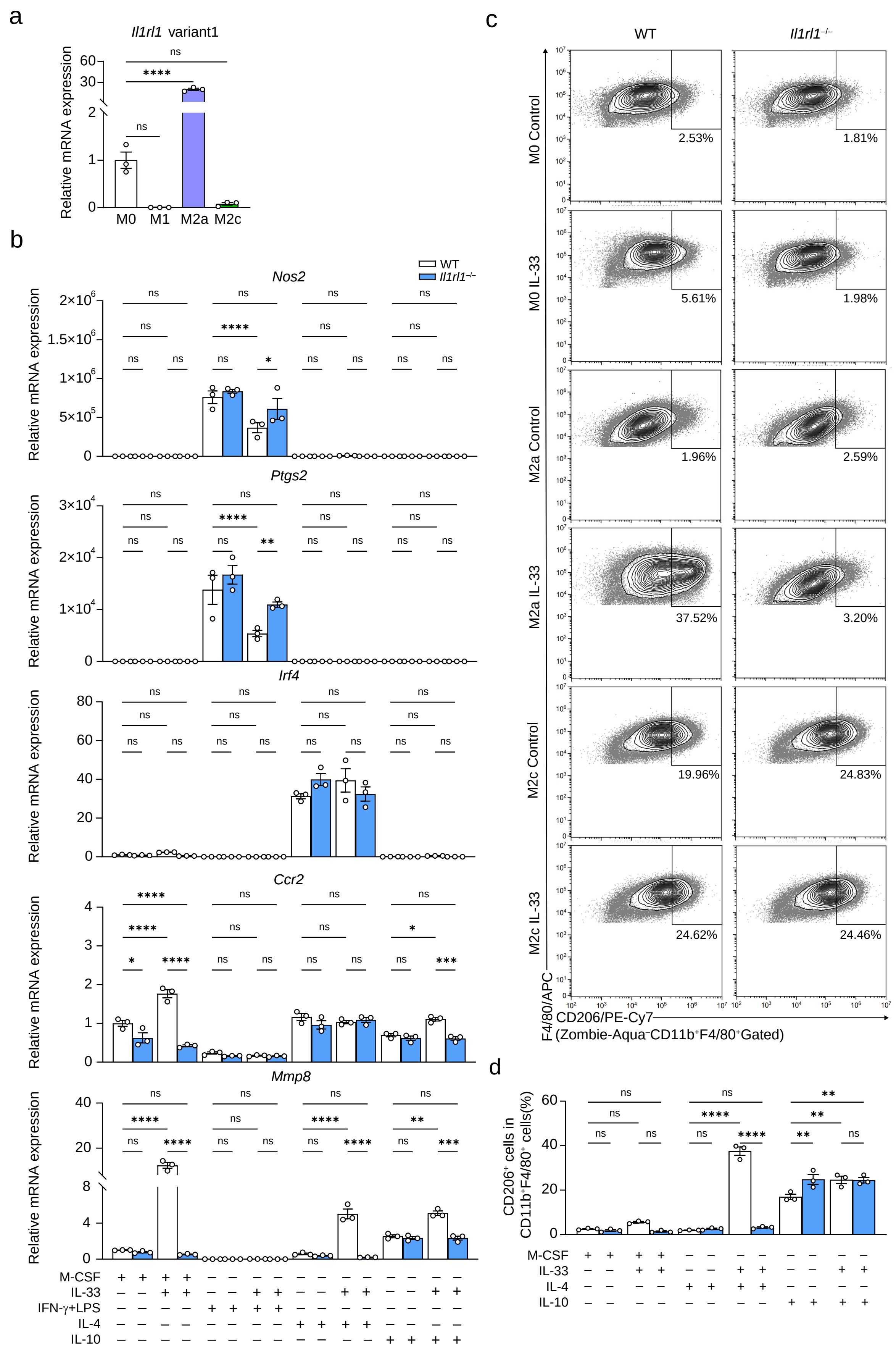

**Supplementary Fig. 28 The impact of IL-33 on macrophage polarization. | a** mRNA expression of *Il1rl1* variant 1 (mST2) of the normally polarized macrophages differentiated from the BMDM of WT mice *in vitro* (M0 macrophage = 1; *n* = 3 mice per group). **b** mRNA expression of *Nos2*, *Ptgs2*, *Irf4*, *Ccr2*, and *Mmp8* of the polarized macrophages differentiated from the BMDM of WT and *Il1rl1*$^{-/-}$ mice *in vitro*. WT M0 Control (no treatment with rmIL-33) = 1; *n* = 3 mice per group. **c** Changes in CD206 expression of IL-33-treated/nontreated M0/M2a/M2c polarized macrophages differentiated from the BMDM of WT and *Il1rl1*$^{-/-}$ mice *in vitro*. A representative contour plot is shown, and the percentages shown in the gates are the mean of three individual mouse experiments. **d** Changes in the cell percentages (in CD11b$^{+}$F4/80$^{+}$ cells) of CD206$^{+}$ cells in M0/M2a/M2c polarized macrophages with the treatment of rmIL-33 (*n* = 3 mice per group). Data are presented as the mean ± SEM. *P < 0.05; **P < 0.01; ns, P > 0.05; by one-way ANOVA with multiple comparisons via Dunnett's test (**a**); and by two-way ANOVA with multiple comparisons via Tukey's test (**b** and **d**). The exact P values are shown in Supplementary Data. 2. The related gating strategy was shown in Supplementary Fig. 33b (**c, d**). Source data are provided as a Source Data file.

Supplementary Fig. 29

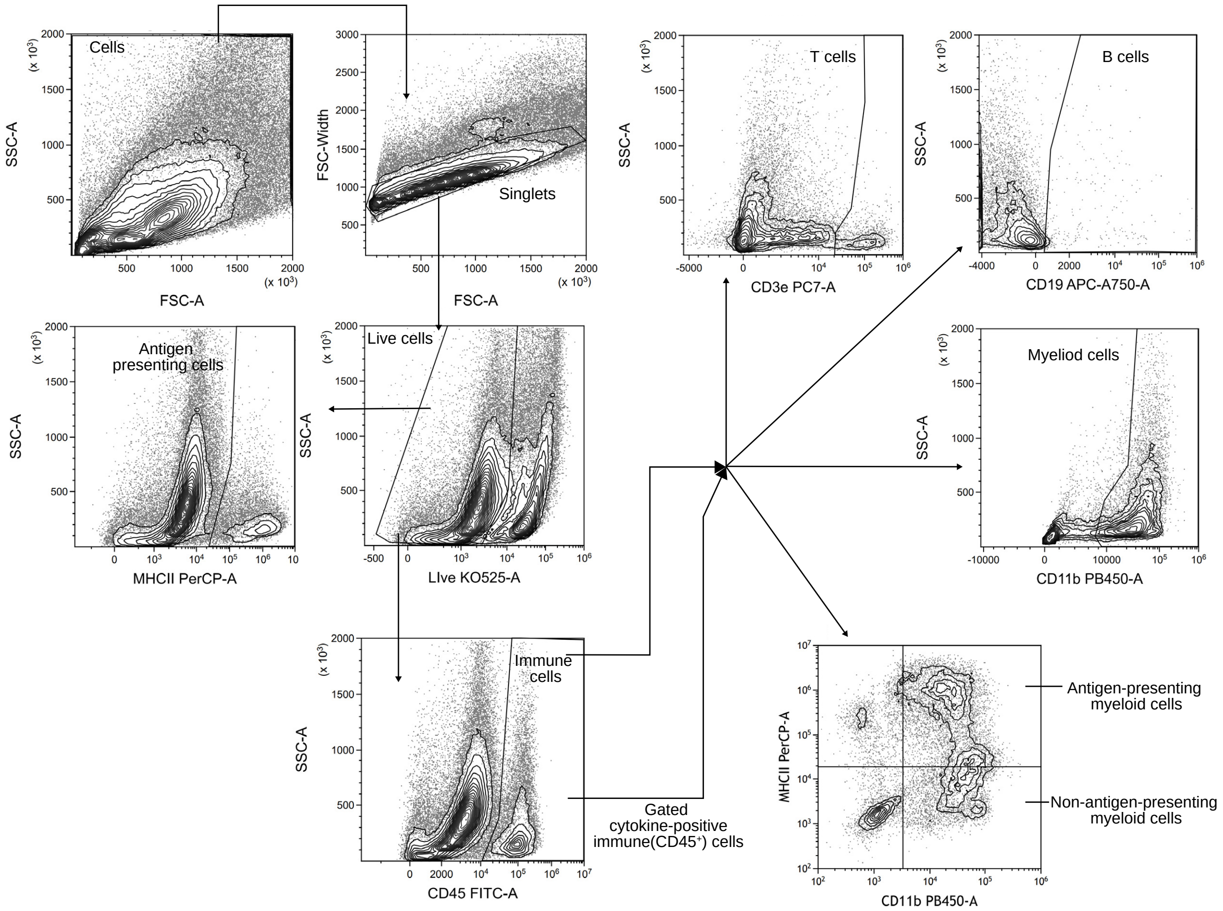


**Supplementary Fig. 29 Gating strategy for general analysis (Strategy 1). |** Gating strategy for general analysis was shown using representative data of peri-odontitis-induced peri-root tissue. The details are described in the Reporting Summary.

Supplementary Fig. 30

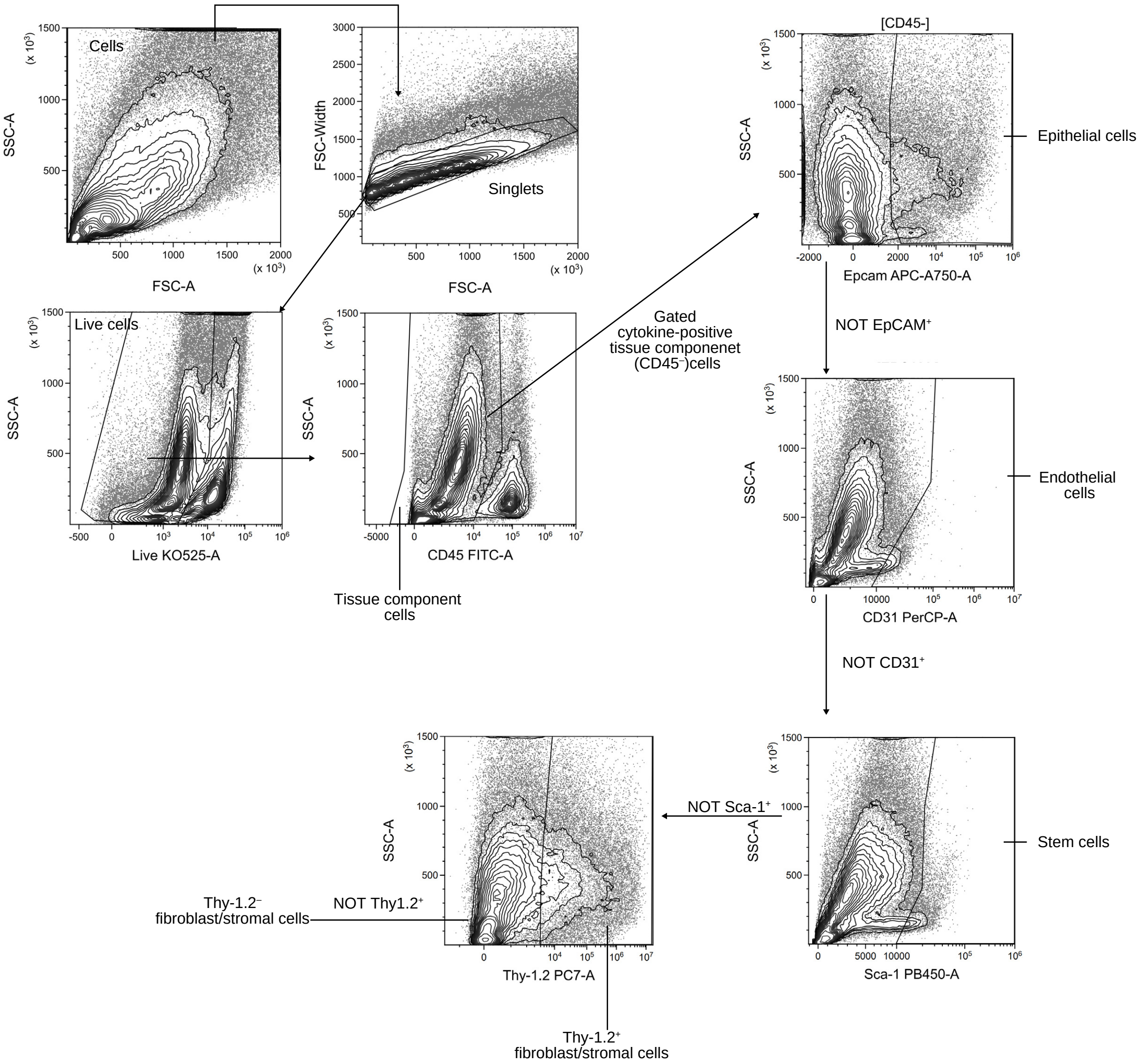


**Supplementary Fig. 30 Gating strategy for tissue component cells (Strategy 2). |** Gating strategy for tissue component cells was shown using representative data of periodontitis-induced peri-root tissue. The details are described in the Reporting Summary.

Supplementary Fig. 31

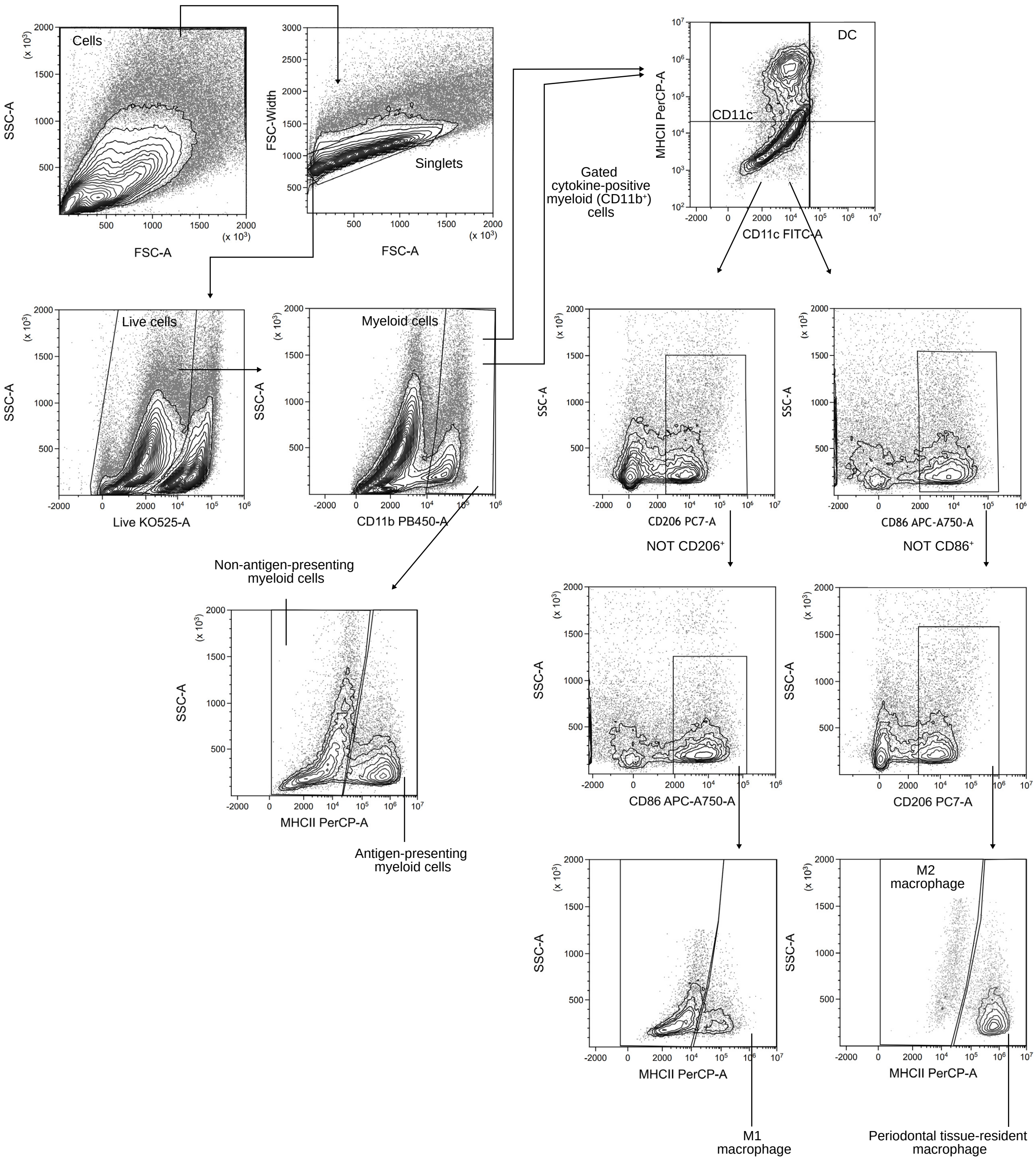


**Supplementary Fig. 31 Gating strategy for antigen-presenting myeloid cells (Strategy 3). |** Gating strategy for antigen-presenting myeloid cells was shown using representative data of periodontitis-induced peri-root tissue. The details are described in the Reporting Summary.

Supplementary Fig. 32

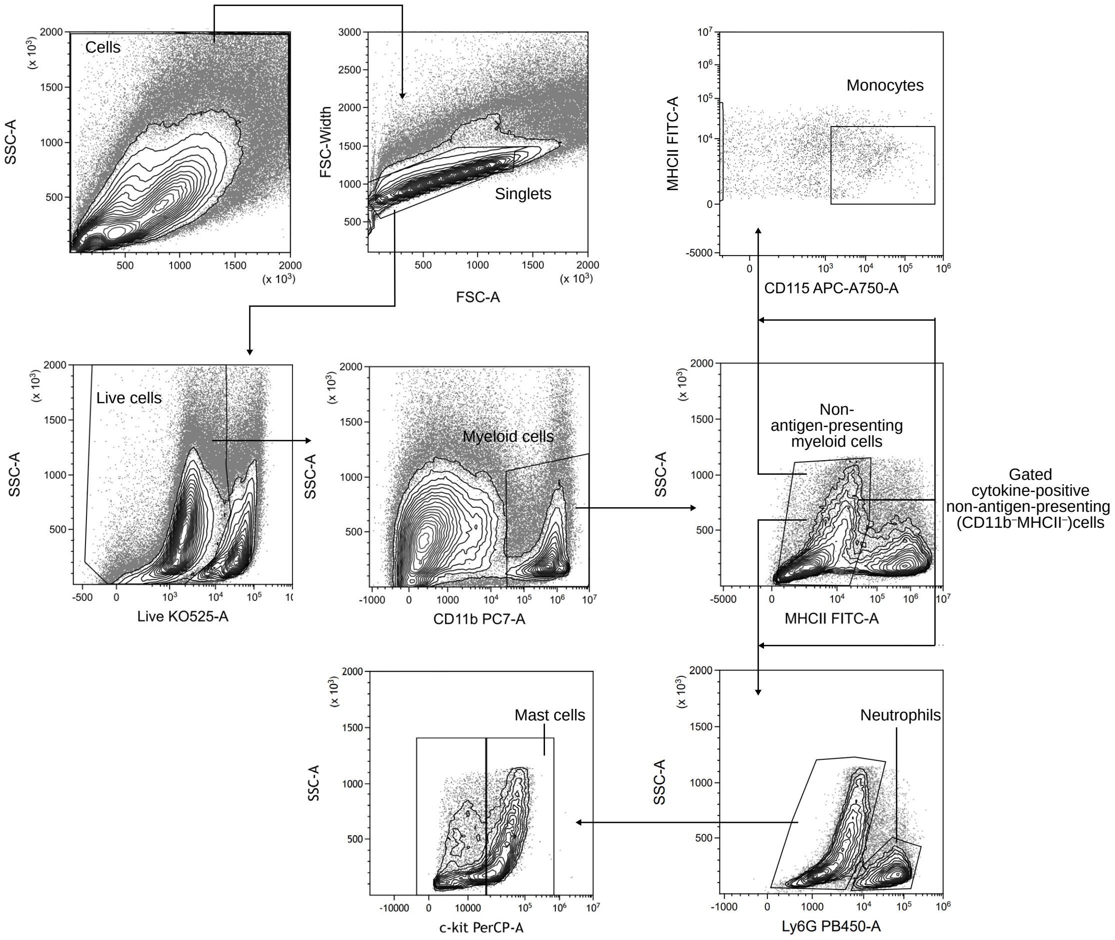


**Supplementary Fig. 32 Gating strategy for non-antigen-presenting myeloid cells (Strategy 4). |** Gating strategy for non-antigen-presenting myeloid cells was shown using representative data of periodontitis-induced peri-root tissue. The details are described in the Reporting Summary.

Supplementary Fig. 33

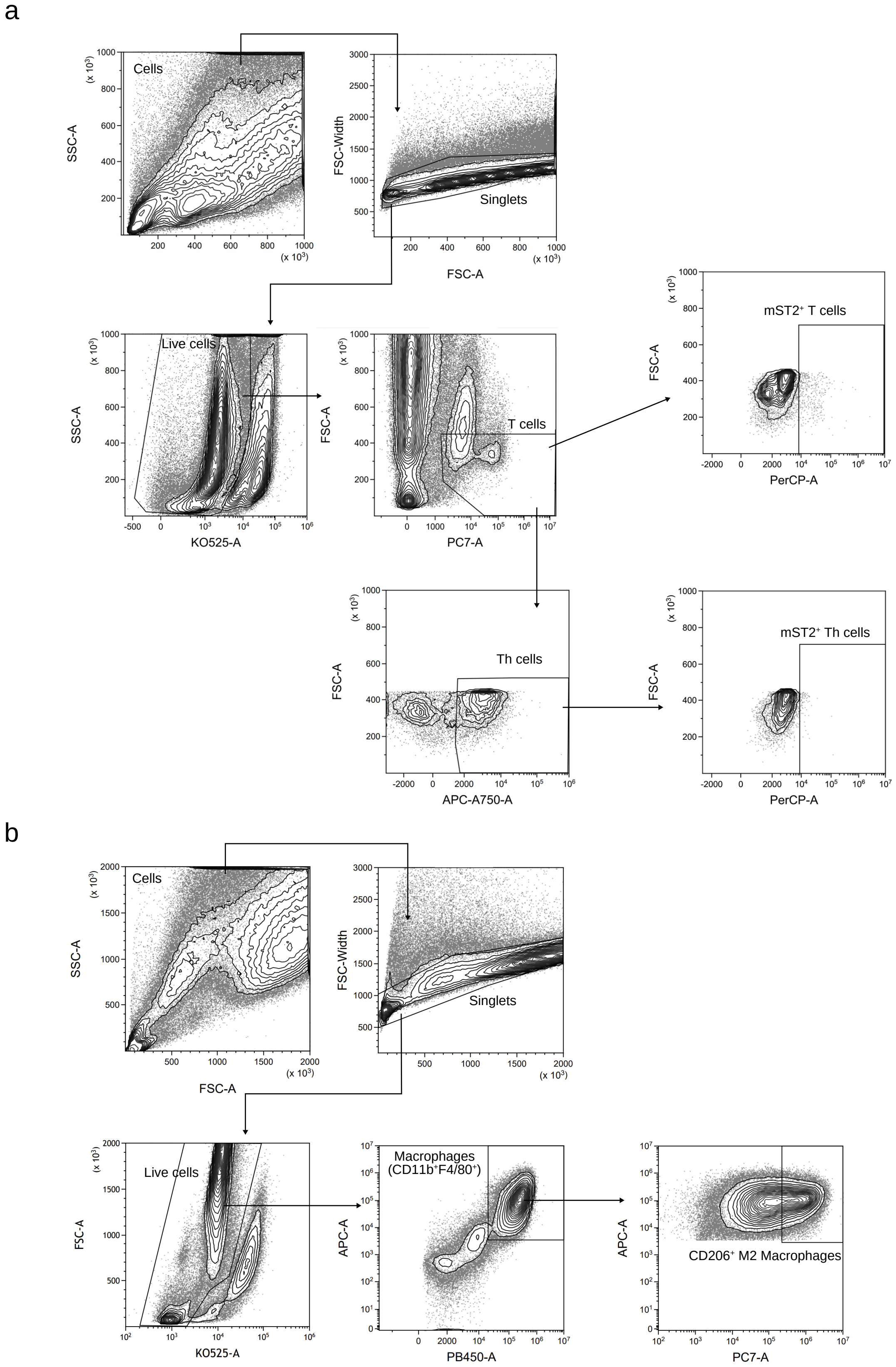


**Supplementary Fig. 33 Gating strategy for CD4$^+$ T cells and evaluation of M2 polarization *in vitro*. | a** Gating strategy for CD4$^+$ T cells (Strategy 5) was shown using representative data of periodontitis-induced peri-root tissue. **b** Gating strategy for evaluating M2 polarization was shown using representative data of IL-33 treated M2a-polarized bone marrow-delivered macrophages differentiated from WT mice. The details are described in the Reporting Summary.

Supplementary Table. 1

| Direction | GSEA analysis: KOPL vs WTPL | NES | Genes | adj.Pval |
|---|---|---|---|---|
| Down | Regulation of glucocorticoid secretion | -0.9525 | 6 | 8.30E-03 |
| | Negative regulation of steroid hormone secretion | -0.93 | 5 | 4.80E-02 |
| | Negative regulation of corticosteroid hormone secretion | -0.93 | 5 | 4.80E-02 |
| | Negative regulation of glucocorticoid secretion | -0.93 | 5 | 4.80E-02 |
| | Regulation of antimicrobial peptide production | -0.9085 | 5 | 1.30E-01 |
| | ERBB4 signaling pathway | -0.9015 | 5 | 1.50E-01 |
| | Cornification | -0.8996 | 5 | 1.60E-01 |
| | Regulation of antimicrobial humoral response | -0.8991 | 7 | 3.50E-02 |
| | Myosin filament organization | -0.8959 | 5 | 1.60E-01 |
| | Positive regulation of T-helper 17 cell differentiation | -0.8938 | 5 | 1.70E-01 |
| | Skeletal myofibril assembly | -0.8925 | 6 | 1.20E-01 |
| | Positive regulation of thymocyte apoptotic process | -0.8862 | 6 | 1.40E-01 |
| | Positive regulation of neutrophil activation | -0.8823 | 6 | 1.50E-01 |
| | Glucocorticoid secretion | -0.8739 | 8 | 1.00E-01 |
| | Regulation of macrophage proliferation | -0.865 | 6 | 1.90E-01 |
| | ERBB2 signaling pathway | -0.8633 | 10 | 2.30E-02 |
| | ERBB2-EGFR signaling pathway | -0.8607 | 7 | 1.50E-01 |
| | Regulation of skeletal muscle contraction | -0.8508 | 9 | 1.00E-01 |
| | Natural killer cell activation involved in immune response | -0.8498 | 10 | 3.20E-02 |
| | Enucleate erythrocyte differentiation | -0.8496 | 9 | 1.10E-01 |
| | Branching involved in labyrinthine layer morphogenesis | -0.8401 | 9 | 1.20E-01 |
| | Positive regulation of epidermal growth factor-activated receptor activity | -0.8382 | 12 | 2.40E-02 |
| | Positive regulation of humoral immune response | -0.8275 | 12 | 3.20E-02 |
| | Positive regulation of interleukin-4 production | -0.8269 | 17 | 7.40E-03 |
| | Mucosal immune response | -0.8197 | 12 | 3.60E-02 |
| | Innate immune response in mucosa | -0.8183 | 10 | 1.40E-01 |
| Up | Response to iron(II) ion | 0.9313 | 6 | 1.10E-02 |
| | Regulation of SNARE complex assembly | 0.9226 | 5 | 4.10E-02 |
| | Retinal rod cell development | 0.8499 | 6 | 1.60E-01 |
| | Response to folic acid | 0.8265 | 7 | 1.40E-01 |

**The details of the GESA analysis results of Supplementary Fig. 23a.** Upregulated and downregulated pathways enriched in PRT on day 11 are showed with NES values and included gene numbers, and sorted according to the adjusted P-values. PRT: peri-root tissue. P-values are adjusted by Benjamini & Hochberg method.

Supplementary Table. 2

| Direction | GSEA analysis: KOPL vs WTPL | NES | Genes | adj.Pval |
| --- | --- | --- | --- | --- |
| Down | Steroid biosynthesis | -0.822 | 14 | 2.00E-02 |
|  | Retinol metabolism | -0.6602 | 26 | 1.90E-01 |
|  | Staphylococcus aureus infection | -0.6445 | 56 | 1.10E-02 |
|  | C-type lectin receptor signaling pathway | -0.5602 | 92 | 1.10E-02 |
|  | IL-17 signaling pathway | -0.5535 | 71 | 4.50E-02 |
|  | Neutrophil extracellular trap formation | -0.5438 | 109 | 1.10E-02 |
|  | Acute myeloid leukemia | -0.5333 | 62 | 1.90E-01 |
|  | NF-kappa B signaling pathway | -0.512 | 95 | 4.50E-02 |
|  | Amoebiasis | -0.5105 | 79 | 1.60E-01 |
|  | Rheumatoid arthritis | -0.5091 | 69 | 1.90E-01 |
|  | Cytokine-cytokine receptor interaction | -0.5066 | 167 | 1.10E-02 |
|  | Leukocyte transendothelial migration | -0.4979 | 91 | 1.60E-01 |
|  | JAK-STAT signaling pathway | -0.4673 | 109 | 1.60E-01 |
|  | Calcium signaling pathway | -0.4439 | 145 | 1.60E-01 |
|  | Tight junction | -0.4378 | 135 | 1.90E-01 |

**The details of the GESA analysis results of Supplementary Fig. 23b.** Downregulated pathways enriched in the PRT on day 11 are showed with NES values and included gene numbers, and sorted according to the adjusted P-values. PRT: peri-root tissue. P-values are adjusted by Benjamini & Hochberg method.

Supplementary Table. 3

| Direction | GSEA analysis: KOBL vs WTBL | NES | Genes | adj.Pval |
|---|---|---|---|---|
| Down | Positive regulation of cAMP-mediated signaling | -0.8772 | 16 | 6.90E-02 |
| | Sarcoplasmic reticulum calcium ion transport | -0.7927 | 31 | 3.30E-02 |
| | Skeletal muscle contraction | -0.7607 | 42 | 1.60E-02 |
| | Multicellular organismal movement | -0.726 | 55 | 1.70E-02 |
| | Musculoskeletal movement | -0.726 | 55 | 1.70E-02 |
| | Striated muscle adaptation | -0.7187 | 45 | 9.40E-02 |
| | Striated muscle contraction | -0.5877 | 156 | 5.20E-03 |
| | Muscle adaptation | -0.5807 | 116 | 5.10E-02 |
| | Cilium movement | -0.5515 | 155 | 2.80E-02 |
| | Muscle cell development | -0.55 | 192 | 6.60E-03 |
| | Skeletal muscle organ development | -0.5132 | 177 | 6.40E-02 |
| Up | Positive regulation of tooth mineralization | 0.9829 | 5 | 3.20E-02 |
| | Regulation of enamel mineralization | 0.982 | 5 | 3.60E-02 |
| | Regulation of tooth mineralization | 0.9511 | 8 | 3.60E-02 |
| | Response to aldosterone | 0.9297 | 12 | 1.10E-02 |
| | Cellular response to mineralocorticoid stimulus | 0.9197 | 11 | 2.90E-02 |
| | Enamel mineralization | 0.889 | 18 | 1.10E-02 |
| | Amelogenesis | 0.8742 | 23 | 6.30E-03 |
| | Cellular response to aldehyde | 0.8725 | 16 | 6.50E-02 |
| | Tooth mineralization | 0.8328 | 28 | 1.10E-02 |
| | Wound healing spreading of cells | 0.7794 | 36 | 4.20E-02 |
| | Epiboly involved in wound healing | 0.7794 | 36 | 4.20E-02 |
| | Epiboly | 0.7732 | 37 | 2.80E-02 |
| | Vitamin transport | 0.7679 | 35 | 2.80E-02 |
| | Regulation of water loss via skin | 0.7637 | 32 | 9.20E-02 |
| | Multicellular organismal water homeostasis | 0.759 | 55 | 5.20E-03 |
| | Cellular response to interferon-beta | 0.7523 | 51 | 6.60E-03 |
| | Water homeostasis | 0.7504 | 59 | 3.00E-03 |
| | Response to interferon-beta | 0.7308 | 60 | 6.60E-03 |
| | Odontogenesis | 0.5794 | 120 | 1.00E-01 |

**The details of the GESA analysis results of Supplementary Fig. 23c.**
Upregulated and downregulated pathways enriched in the BT on day 11 are showed with NES values and included gene numbers, and sorted according to the adjusted P values. BT: bone tissue. P-values are adjusted by Benjamini & Hochberg method.

Supplementary Table. 4

| mRNA | Primer sequence(5'-3') | Product length(bp) |
|---|---|---|
| ***Il1b*** | F:TGCCACCTTTTGACAGTGATG<br>R:ATGTGCTGCTGCGAGATTTG | 136 |
| ***Il6*** | F:CGGCCTTCCCTACTTCACAA<br>R:GAATTGCCATTGCACAACTCT | 154 |
| ***Il10*** | F:GGCCCAGAAATCAAGGAGCA<br>R:AGACACCTTGGTCTTGGAGCTTA | 160 |
| ***Il17a*** | F:TCCCTCTGTGATCTGGGAAG<br>R:AGCATCTTCTCGACCCTGAA | 162 |
| ***Il33*** | F:AAAGACCAAGAGCAAGACCAGG<br>R:ACCGTCGCCTGATTGACTTG | 75 |
| ***Il33* variant 1** | F:TCCTGTCTGTATTGAGAAACCTGA<br>R:CCGGGGAAATCTTGGAGTTGG | 70 |
| ***Il33* variant 2** | F:CACGGCAGAATCATCGAGAAACC<br>R:TGCCGGGGAAATCTTGGAGT | 74 |
| ***Il1rl1*** | F:AAAGCCGCCAAGCTGCAATA<br>R:GGAGAGCTTTGCAGTTCTTAAACC | 146 |
| ***Il1rl1* variant 1** | F:TTGTTCACCACACTCTGCCC<br>R:ACGGTGGCTGCATCTTGC | 100 |
| ***Il1rl1* variant 1/2** | F:CTCCAGCCCTTCATCTGGGTATCTA<br>R:TGGTGTGTTCACTAGGCGGTTG | 88 |
| ***Tnf*** | F:CCAGACCCTCACACTCAGATCA<br>R:GTCTTTGAGATCCATGCCGTTGG | 148 |
| ***Tnfsf11*** | F:GCAGAAGGAACTGCAACACA<br>R:GATGGTGAGGTGTGCAAATG | 130 |
| ***Tnfrsf11b*** | F:GTTTCCCGAGGACCACAAT<br>R:CCATTCAATGATGTCCAGGAG | 71 |
| ***Krt5*** | F:AGATGTTCTTTGATGCGGAGC<br>R:TGTCCATGGAAAGGACCACAG | 75 |
| ***Dsc2*** | F:AGGGCCCAGTAGAGGTACTAA<br>R:AGCCCATCTTCTCTTGGCAC | 74 |
| ***Postn*** | F:AGACTGCTTCAGGGAGACAC<br>R:ACGGCCTTCTCTTGATCGTC | 87 |
| ***Aspn*** | F:AGTCTTGAGGATACTTACATTGCAG<br>R:CCTGTGTACAAGTCCAACCTTGTC | 101 |
| ***Sp7*** | F:ATGGCGTCCTCTCTGCTTG<br>R:TGAAAGGTCAGCGTATGGCTT | 156 |
| ***Bglap*** | F:CTGACCTCACAGATGCCAAGC<br>R:TGGTCTGATAGCTCGTCACAAG | 187 |
| ***Eef2*** | F:TCACAATCAAATCCACCGCCA<br>R:AGGTTGATGAGGAAGCCCGA | 103 |

**Summary of the primer sets and product lengths used for RT-qPCR analysis in this study (tissue samples).**

The primers are designed by primer-BLAST of NCBI (https://www.ncbi.nlm.nih.gov/tools/primer-blast/) up to March 11th, 2023. All product length are confirmed.

Supplementary Table. 5

| mRNA | Primer sequence(5'-3') | Product length(bp) |
|---|---|---|
| ***Nos2*** | F:GGTACGCTGGCTACCAGATG | 80 |
| | R:GTCGATGCACAACTGGGTGA | |
| ***Ptgs2*** | F:AAGGCCTCCATTGACCAGAG | 121 |
| | R:CAGCCATTTCCTTCTCTCCTGTA | |
| ***Arg1*** | F:CATTGGCTTGCGAGACGTAG | 151 |
| | R:CGGCCTTTTCTTCCTTCCCAG | |
| ***Irf4*** | F:GTTGCCAGGTGACAGGAACC | 124 |
| | R:ATATGCAGCCGGCAGTCTGAG | |
| ***Ccr2*** | F:AGTTCAGCTGCCTGCAAAGA | 150 |
| | R:GAGATGTTGATAGTATGCCGTGG | |
| ***Mmp8*** | F:AACAACCAGTGCTGGAGATATGA | 82 |
| | R:CTGGGAACATGCTTGGTATGC | |
| ***Stx5a*** | F:AAGCGCAAGTCCCTCTTTGA | 196 |
| | R:AAGCCAGCTTTGACTGCAAC | |

**Summary of the primer sets and product lengths used for RT-qPCR analysis in this study (macrophage polarization experiments).**
The primers are designed by primer-BLAST of NCBI (https://www.ncbi.nlm.nih.gov/tools/primer-blast/) before December 7th, 2023. All product length are confirmed.

**Generation of mice carrying the *Il33* conditional allele.**

All fragments for the construction of the *Il33* targeting vector were derived from a BAC clone (RP23-381K18: BACPAC Resource Center) by restriction digestion and subcloned into a pBluescript II vector. The homology arms were cloned into a targeting vector backbone containing PGK-*neo* and DTA-positive and -negative selection cassettes (pLFNeo-DTA vector) (Supplementary Fig. 20b). The long arm was excised as a *NcoI/EcoRV* fragment (5.5 kb) from the pBluescript II vector including all of the homology arms of *Il33*, and cloned into the *SacII* sites of a pLFNeo-DTA vector upstream of the *neo* selection cassette, which was flanked by *frt* sites. For construction of the middle arm, a fragment encompassing exon 5 to 7 of *Il33* was excised as the *EcoRV/AhdI* fragment (3.4 kb), and cloned into the *NheI* site of the pLFNeo-DTA vector just upstream of the *neo* selection cassette so as to flank exon 5 to 7 with *loxP* sites. The short arm was excised as the *AhdI/SacI* fragment (0.5 kb), then cloned into the *EcoRV* sites of the pLFNeo-DTA vector upstream of the DTA cassette to give rise to the final targeting vector for the *Il33*$^{\mathrm{floxNeo}}$ allele. The targeting vector was linearized with *XhoI* and electroporated into A9 embryonic stem (ES) cells. A9 ES cells were derived from the 129 and C57BL/6 hybrid strain (Nakashima et al. *Nat Med* 2011). After 8–9 days of G418 selection, resistant single colonies were picked and transferred onto primary embryonic fibroblast cells acting as feeder cells, and expanded to allow diagnosis of homologous recombination. Potential recombinant clones were detected by genomic PCR. The forward primer (PS1: the construct-specific primer, 5’-AGACTGCCTTGGGAAAAG-3’) binds within the *neo* selection cassette region, the reverse primer (PS2: the locus-specific primer, 5’-CCCTGCCAATGAATACTG-3’) binds to a unique region outside the short arm of the targeting vector and within the endogenous locus. The *loxP* site between the long and middle arms was confirmed using the primer pairs (P1 and P2: described below). Approximately 2–3 out of 100 ES cell clones were identified as correctly targeted by genomic PCR. Targeted ES cells were injected into C57BL/6J blastocysts to achieve initial germ-line transmission. Chimeric male mice were crossed with C57BL/6J females to establish a line for the *Il33*$^{\mathrm{floxNeo}}$ allele. Mice carrying the *frt*-flanked PGK-*neo* cassette was crossed with C57BL/6J background FLPe transgenic mice to excise the PGK-*neo* cassette and subsequently crossed to C57BL/6J mice to remove the FLPe recombinase transgene so as to generate *Il33*$^{\mathrm{flox}}$ mice. In parallel, *Il33*$^{\mathrm{flox}}$ mice were also crossed to C57BL/6J background *Actb-Cre* ubiquitous deleter mice to generate mice carrying a *Il33*$^{\Delta}$ allele (Global KO). Mice carrying the *Il33*$^{\mathrm{flox}}$ or *Il33*$^{\Delta}$ alleles were backcrossed more than 7 generations to C57BL/6J mice. These mice were then intercrossed to generate *Il33*$^{\mathrm{flox}/\Delta}$

and *Il33*$^{+/\Delta}$ mice. For mouse genotyping, genomic DNA from mouse tails was isolated by phenol/chloroform extraction and ethanol precipitation, and amplified by PCR with three primers: P1, 5'-ACATAGTTCTGCACGCTG-3'; P2, 5'-GTCCAGACCTTTTGTGGG-3'; and P3, described above. The PCR products were 160 bp (wild-type allele, primers P1/P2), 291 bp (floxed allele, primers P1/P2) and 546 bp (delta allele, primers P1/P3), showed in supplementary figure (Supplementary Fig. 20c). The *Cre* transgene was detected as a 455 bp PCR product by using the forward primer 5'-TCGCGATTATCTTCTATATCTTCAG-3' and reverse primer 5'-GCTCGACCAGTTTAGTTACCC-3'. The expression loss of normal *Il33* mRNA was further confirmed with RT-qPCR in periodontal tissues before use (Supplementary Fig. 20d).
