# Supplementary material for: The IL-33/ST2 axis is protective against acute inflammation during the course of periodontitis
Source: Nat Commun. 2024 Mar 28;15:2707. doi: 10.1038/s41467-024-46746-2 (PMC10978877; doi:10.1038/s41467-024-46746-2)
Supplement: Supplementary file 3 — Description of Additional Supplementary Files [file 41467_2024_46746_MOESM3_ESM.pdf]

Description/Legends of Supplementary files

File Name: Supplementary Data. 1
Description: Summary of the P-values featured in the graphs of the main figures. The values are categorized into separate sheets based on the corresponding figure numbers and sorted according to their position in the figure.

File Name: Supplementary Data. 2
Description: Summary of the P-values featured in the graphs of the supplementary figures. The values are categorized into separate sheets based on the corresponding figure numbers and sorted according to their position in the figure.

File Name: Supplementary Movie. 1
Description: Instructions for inducing a modified ligature model in mice. Place the ligature between the molars and adjust it to fit the external part of the second molar. Then, form two loops with the ligature, insert it again between the molars and adjust it to fit the cervical parts of the first and third molars. Finally, tie a knot to secure it in place (not demonstrated in the video).
