# Supplementary material for: The IL-33/ST2 axis is protective against acute inflammation during the course of periodontitis
Source: Nat Commun. 2024 Mar 28;15:2707. doi: 10.1038/s41467-024-46746-2 (PMC10978877; doi:10.1038/s41467-024-46746-2)
Supplement: Supplementary file 7 — Reporting Summary [file 41467_2024_46746_MOESM7_ESM.pdf]

nature portfolio

Corresponding author(s): Tomoki Nakashima

Last updated by author(s): Feb 25, 2024

Reporting Summary

Nature Portfolio wishes to improve the reproducibility of the work that we publish. This form provides structure for consistency and transparency in reporting. For further information on Nature Portfolio policies, see our Editorial Policies and the Editorial Policy Checklist.

Statistics

For all statistical analyses, confirm that the following items are present in the figure legend, table legend, main text, or Methods section.

n/a	Confirmed	
☐	☒	The exact sample size (*n*) for each experimental group/condition, given as a discrete number and unit of measurement
☐	☒	A statement on whether measurements were taken from distinct samples or whether the same sample was measured repeatedly
☐	☒	The statistical test(s) used AND whether they are one- or two-sided<br>*Only common tests should be described solely by name; describe more complex techniques in the Methods section.*
☒	☐	A description of all covariates tested
☐	☒	A description of any assumptions or corrections, such as tests of normality and adjustment for multiple comparisons
☐	☒	A full description of the statistical parameters including central tendency (e.g. means) or other basic estimates (e.g. regression coefficient) AND variation (e.g. standard deviation) or associated estimates of uncertainty (e.g. confidence intervals)
☐	☒	For null hypothesis testing, the test statistic (e.g. *F*, *t*, *r*) with confidence intervals, effect sizes, degrees of freedom and *P* value noted<br>*Give P values as exact values whenever suitable.*
☒	☐	For Bayesian analysis, information on the choice of priors and Markov chain Monte Carlo settings
☒	☐	For hierarchical and complex designs, identification of the appropriate level for tests and full reporting of outcomes
☒	☐	Estimates of effect sizes (e.g. Cohen's *d*, Pearson's *r*), indicating how they were calculated

Our web collection on statistics for biologists contains articles on many of the points above.

Software and code

Policy information about availability of computer code

Data collection	CytExpert v2.3.1.22 (Beckman Coulter), BZ-X_Analyzer v.1.4.0.1 (Keyence), inspeXio v7.2.2.1695 (SHIMADZU), Bio-Rad CDX Manager v3.11517.0823 (Bio-Rad).
Data analysis	Kaluza v2.1 (Beckman Coulter), Graph Pad Prism v9/10 (Graph Pad Prism Software), TRI/3D-BON-FCS v10.01.37.47-H-64 (RATOC), Trimmomatic v0.39, FastQC v0.11.9, HISAT2 v2.2.1, StringTie v2.1.7, DESeq2 v1.32.0, ImmuCC(https://github.com/wuaipinglab/ImmuCC), iDEP v1.1.1(http://bioinformatics.sdstate.edu/idep11/).

For manuscripts utilizing custom algorithms or software that are central to the research but not yet described in published literature, software must be made available to editors and reviewers. We strongly encourage code deposition in a community repository (e.g. GitHub). See the Nature Portfolio guidelines for submitting code & software for further information.

Data

Policy information about availability of data

All manuscripts must include a data availability statement. This statement should provide the following information, where applicable:

- Accession codes, unique identifiers, or web links for publicly available datasets
- A description of any restrictions on data availability
- For clinical datasets or third party data, please ensure that the statement adheres to our policy

The RNAseq data produced in this study were deposited to the public database (GSE221720: https://www.ncbi.nlm.nih.gov/geo/query/acc.cgi?acc=GSE221720;

GSE244931: https://www.ncbi.nlm.nih.gov/geo/query/acc.cgi?acc=GSE244931). We also used the mouse transcriptome index mus musculus GRCm39 (https://www.ncbi.nlm.nih.gov/datasets/genome/GCF_000001635.27/) and KEGG annotation data in Hiplot platform (https://download.hiplot-academic.com/api/file/fetch/?path=/f1b0ff00-3d9a-11ed-9ee1-85e3cd828dce/public/db/kegg/mmu_kegg_20220421.rds). Source data are provided with this paper.

Research involving human participants, their data, or biological material

Policy information about studies with human participants or human data. See also policy information about sex, gender (identity/presentation), and sexual orientation and race, ethnicity and racism.

Reporting on sex and gender	NA
Reporting on race, ethnicity, or other socially relevant groupings	NA
Population characteristics	NA
Recruitment	NA
Ethics oversight	NA

Note that full information on the approval of the study protocol must also be provided in the manuscript.

Field-specific reporting

Please select the one below that is the best fit for your research. If you are not sure, read the appropriate sections before making your selection.

☒ Life sciences ☐ Behavioural & social sciences ☐ Ecological, evolutionary & environmental sciences

For a reference copy of the document with all sections, see nature.com/documents/nr-reporting-summary-flat.pdf

Life sciences study design

All studies must disclose on these points even when the disclosure is negative.

Sample size	No sample-size calculation was performed. The sample sizes were guided by previous studies using similar analyses (Tsukasaki, M et al., Nature Communications, 2018).
Data exclusions	RT-qPCR data were examined using Grubb's test and outliers were excluded with the criterion of alpha = 0.05.
Replication	The number of biological replicates (n = 3 for flow cytometry, immunofluorescence staining, and RT-qPCR of cell samples; n = 4 for µCT analyses, RT-qPCR of tissue samples, RNA-Sequencing, and histological experiments) was detailed in Methods section and the corresponding figure legends. There were some replicates that were considered irregular and excluded in RT-qPCR of tissue samples using Grrub's test as described above.
Randomization	Due to the experiment mainly being performed with a split-mouse design, only assigning animals to different ligature methods groups or experiment/control groups in flow cytometory within wild-type mice and assigning mice to different time points within same genotype are randomized in this study.
Blinding	Investigators were not blinded to group allocation during data collection or analysis. Blinding were not relevant to this study because the groups are defined by genotype and ligature placement methods.

Reporting for specific materials, systems and methods

We require information from authors about some types of materials, experimental systems and methods used in many studies. Here, indicate whether each material, system or method listed is relevant to your study. If you are not sure if a list item applies to your research, read the appropriate section before selecting a response.

Materials & experimental systems

n/a	Involved in the study
☐	☒ Antibodies
☒	☐ Eukaryotic cell lines
☒	☐ Palaeontology and archaeology
☐	☒ Animals and other organisms
☒	☐ Clinical data
☒	☐ Dual use research of concern
☒	☐ Plants

Methods

n/a	Involved in the study
☒	☐ ChIP-seq
☐	☒ Flow cytometry
☒	☐ MRI-based neuroimaging

Antibodies

Antibodies used

1. Pacific Blue™ anti-mouse/human CD11b (M1/70, BioLegend, Cat# 101223, 1:200 dilution for FCM)
2. Pacific Blue™ anti-mouse Ly-6A/E (Sca-1) (D7, BioLegend, Cat# 108119, 1:200 dilution for FCM)
3. Pacific Blue™ anti-mouse Ly-6G Antibody (1A8, BioLegend, Cat# 127611, 1:100 dilution for FCM)
4. FITC anti-mouse CD11c (N418, BioLegend, Cat# 1173051, 1:100 dilution for FCM)
5. FITC anti-mouse CD45 (30-F11, BioLegend, Cat# 103107, 1:200 dilution for FCM)
6. FITC anti-mouse I-A/I-E (M5/114.15.2, BioLegend, Cat# 107605, 1:200 dilution for FCM)
7. PE anti-mouse CD254 (TRANCE, RANKL) (IK22/5, BioLegend, Cat# 510005, 1:100 dilution for intracellular FCM)
8. PE anti-mouse IL-6 (MP5-20F3, BioLegend, Cat# 504503, 1:100 dilution for intracellular FCM)
9. PE anti-mouse IL-33Rα (IL1RL1, ST2) (DIH9, BioLegend, Cat# 145303, 1:100 dilution for intracellular FCM)
10. PerCP/Cyanine5.5 anti-mouse CD31 (390, BioLegend, Cat# 102419, 1:200 dilution for FCM)
11. PerCP/Cyanine5.5 anti-mouse CD117 (c-kit) (2B8, BioLegend, Cat# 105823, 1:100 dilution for FCM)
12. PerCP/Cyanine5.5 anti-mouse I-A/I-E (M5/114.15.2, BioLegend, Cat# 107625, 1:400 dilution for FCM)
13. PerCP/Cyanine5.5 anti-mouse IL-33Rα (IL1RL1, ST2) (DIH9, BioLegend, Cat# 107625, 1:50 dilution for FCM)
14. APC anti-mouse F4/80 (BM8, BioLegend, Cat# 123115, 1:200 dilution for FCM)
15. APC anti-mouse IL-10 (JES5-16E3, BioLegend, Cat# 505009, 1:100 dilution for intracellular FCM)
16. APC anti-mouse IL-17A (TC11-18H10.1, BioLegend, Cat# 506915, 1:100 dilution for intracellular FCM)
17. APC anti-mouse IL-33Rα (IL1RL1, ST2) (DIH9, BioLegend, Cat# 145305, 1:50 dilution for FCM, 1:100 dilution for intracellular FCM)
18. Mouse IL-33 Alexa Fluor® 647-conjugated Antibody (396118, R&D Systems, Cat# IC3626R, 1:100 dilution for FCM)
19. PE/Cyanine7 anti-mouse CD3ε (145-2C11, BioLegend, Cat# 100319, 1:200 dilution for FCM)
20. PE/Cyanine7 anti-mouse/human CD11b (M1/70, BioLegend, Cat# 101215, 1:200 dilution for FCM)
21. PE/Cyanine7 anti-mouse CD90.2 (Thy1.2) (30-H12, BioLegend, Cat# 105325, 1:400 dilution for FCM)
22. PE/Cyanine7 anti-mouse CD206 (MMR) Antibody (C068C2, BioLegend, Cat# 141719, 1:100 dilution for FCM)
23. APC/Cyanine7 anti-mouse CD4 (GK1.5, BioLegend, Cat# 100413, 1:100 dilution for FCM)
24. APC/Cyanine7 anti-mouse CD19 (6D5, BioLegend, Cat# 115529, 1:200 dilution for FCM)
25. APC/Cyanine7 anti-mouse CD86 (GL-1, BioLegend, Cat# 105029, 1:100 dilution for FCM)
26. APC/Cyanine7 anti-mouse CD115 (CSF-1R) (AFS98, BioLegend, Cat# 135531, 1:100 dilution for FCM)
27. APC/Cyanine7 anti-mouse CD326 (Ep-CAM) (G8.8, BioLegend, Cat# 118217, 1:200 dilution for FCM)
28. Purified anti-mouse/human CD31 (390, BioLegend, Cat# 102401, 1:100 dilution for immunofluorescence stain)
29. Purified anti-mouse CD45 (30-F11, BioLegend, Cat# 103101, 1:100 dilution for immunofluorescence staining)
30. Purified anti-mouse CD90.2 (Thy-1.2) Antibody (30-H12, BioLegend, Cat# 105301, 1:200 dilution for immunofluorescence staining)
31. Purified anti-mouse CD326 (Ep-CAM) (G8.8, BioLegend, Cat# 118201, 1:50 dilution for immunofluorescence staining)
32. Anti-MMP9 antibody (Polyclonal Rabbit IgG, Abcam plc, Cat# ab38898, 1:300 dilution for immunofluorescence staining)
33. Mouse IL-1 beta /IL-1F2 Antibody (Polyclonal Goat IgG, R&D Systems, Cat# AF-401-SP, 1:500 dilution for immunofluorescence staining)
34. Mouse IL-6 Antibody (Polyclonal Goat IgG, R&D Systems, Cat# AF-406-SP, 1:150 dilution for immunofluorescence staining)
35. Mouse IL-33 Antibody (Polyclonal Goat IgG, R&D Systems, Cat# AF3626, 1:250 dilution for immunofluorescence staining)
36. Mouse ST2/IL-33R Antibody (Polyclonal Goat IgG, R&D Systems, Cat# AF1004-SP, 1:150 dilution for immunofluorescence staining)
37. Human/Mouse TNF-alpha Antibody (Polyclonal Goat IgG, R&D Systems, Cat# AF-410-SP, 1:100 dilution for immunofluorescence staining)
38. Alexa Fluor® 594 Goat anti-rat IgG (minimal x-reactivity) Antibody (Polyclonal Goat IgG, Biolegend, Cat# 405422, 1:100 dilution for immunofluorescence staining)
39. Alexa Fluor® 647 Donkey anti-rabbit IgG (minimal x-reactivity) Antibody (Polyclonal Donkey IgG, Biolegend, Cat# 406414, 1:500 dilution for immunofluorescence staining)
40. Histofine Simple Stain Mouse MAX PO (G) (HRP-labeled amino acid polymer-conjugated polyclonal Rabbit F(ab), Nichirei Bioscience Inc, Cat# 414351, ready to use for immunofluorescence staining)

Validation

All antibodies used here are commercially available. Antibodies for FCM purchased from BioLegend and R&D Systems are quality control tested by immunofluorescent staining with flow cytometric analysis as mentioned on the manufacturer's website. Antibodies for immunohistochemistry purchased from Biolegend and Abcam plc and NIchirei Bioscience Inc are quality control tested for immunohistochemistry as mentioned on the manufacturer's website. Most of the antibodies (IL-6, IL-33, and TNF-alpha) for immunohistochemistry purchased from R&D Systems are quality control tested for immunohistochemistry as mentioned on the manufacturer's website, and some (IL-1 beta /IL-1F2 and ST2/IL-33R) are quality tested but reported for immunohistochemistry in the reference on the manufacturer's website.

Animals and other research organisms

Policy information about studies involving animals; ARRIVE guidelines recommended for reporting animal research, and Sex and Gender in Research

Laboratory animals	C57BL/6J mice were purchased from CLEA Japan. The Il1rl1-deficient mice were generated and kindly provided by Shizuo Akira (Hoshino, K et al., Journal of Experimental Medicine, 1999). The detailed generation process of Il33Delta/Delta mice is described in the supplementary files (Supplementary Fig. 20, Text. 1). Both strains of transgenic mice were maintained on a C57BL/6J background. All mice were maintained in a 23-25 °C and 40-70% humidity-controlled room under specific-pathogen free condition on a 12 h light/dark cycle with ad libitum access to water and standard laboratory chow diet. Mice were randomly allocated into experimental or control groups (for WT mice) and ensure equal age across genotypes. For µCT analyses, RT-qPCR (tissue samples), and histological experiments, 10-12-week-old age-matched male mice were used because these experiments have a range for two weeks. For flow cytometry (tissue samples) and RNA-Sequencing experiments, 10-week-old age-matched male mice were used (experimental/control animals were co-housed in flow cytometric experiments using WT mice). For any experiments using primary cells, 8-12-week-old age-matched male mice were used for harvesting the cell because the characteristics of bone marrow cells does not change much in this range, and we screened monocytes with cytokine stimulation as well.
Wild animals	The study did not involve wild animals.
Reporting on sex	Periodontitis is a broad-existing disease in humans, and its pathogenesis is not considered to be sex or gender dependent, as it is an bacterium-related disease that has little to do with the reproductive system or sex hormones, except in specific situations such as pregnancy (Kinane, D et al., Nat Rev Dis Primers, 2017). Considering that men and women are almost equally affected by the disease (Peres, MA et al., Lancet, 2019), and that the disease was discussed under general conditions in our study, male mice were chosen for convenience to compare with the results of previous studies.
Field-collected samples	The study did not involve samples collected from the field.
Ethics oversight	All experiments were performed with the approval of the Institutional Animal Care and Use Committee/Genetically Modified Organisms Safety Committee of Tokyo Medical and Dental University.

Note that full information on the approval of the study protocol must also be provided in the manuscript.

Plants

Seed stocks	NA
Novel plant genotypes	NA
Authentication	NA

Flow Cytometry

Plots

Confirm that:

☒ The axis labels state the marker and fluorochrome used (e.g. CD4-FITC).

☒ The axis scales are clearly visible. Include numbers along axes only for bottom left plot of group (a 'group' is an analysis of identical markers).

☒ All plots are contour plots with outliers or pseudocolor plots.

☒ A numerical value for number of cells or percentage (with statistics) is provided.

Methodology

Sample preparation	For harvesting the tissue samples, after euthanasia (CO2) of the mice:<br>1. Blood Samples: 500 µl blood was collected from the heart and maintained in a 1.5ml tube containing EDTA (2.7mM for final concentration) until tissue dissolution was finished.<br>2. Maxillary samples: Separated into GT, PRT, and BT under magnification in PBS supplemented with 2% heat-inactivated fetal bovine serum and maintained in DMEM supplemented with 10% heat-inactivated FBS, 100 U ml–1 penicillin, 100 µg ml–1 streptomycin , 0.25 mg ml–1 DNase I and pH 7.0 25 mM HEPES until tissue dissolution.<br><br>For harvesting the cell samples in macrophage polarization experiments:<br>After washing the cells with warm PBS once, cells were treated with the 10 mM EDTA/PBS in CO2 incubator for 20 min at 37

°C.

For tissue dissolution:
1. PBMC: To obtain the PBMC from blood samples, red blood cell lysis was performed with Hybri-Max™ according to the manufacturer's instructions. Cells were centrifuged at 350 × g, washed with 2% FBS/PBS, and filtered with a 70 µm cell strainer to obtain a single-cell suspension.
2. GT: Tissues were incubated in the digestion buffer consisting of DMEM supplemented with 6% heat-inactivated FBS, 100 U ml–1 penicillin, 100 µg ml–1 streptomycin, pH 7.0 25 mM HEPES , 0.15 mg ml–1 DNase I, and 4 or 1 mg ml–1 Dispase II (4 mg ml–1 for analyzing tissue component cells, 1 mg ml–1 for analyzing myeloid cell and T cell subsets) at 37°C for 30 min with 65 rpm horizontal shaking. GT was temporarily removed from the buffer and thoroughly minced after its epithelial layer was peeled off. Then the 1:1 mixture of Collagenase I and Collagenase II was added to a final concentration of 1 mg ml–1 and incubated for another 30 min as previously. The reaction was terminated by adding EDTA for a final concentration of 5 mM. Tissue fragments were centrifuged at 350 × g, washed with 2% FBS/PBS, and filtered with a 70 µm cell strainer to obtain a single-cell suspension.
3. BT: After crushing BT with scissors, tissues were incubated same as GT except for reduction of the peeling step and the addition of red blood cell lysis step same as PBMC.
4. PRT: Tissues were incubated same as GT except for peeling and mincing steps.

For staining cells digested from tissue samples:
Live cells in each sample were counted with Trypan blue staining, and at least 400,000 cells for GT, 200,000 cells for PRT, 1,000,000 cells for BT, and 5,000,000 cells for PMBC were kept for the staining. Before staining, cells were stimulated in a 37° C incubator with Cell Activation Cocktail and Brefeldin A for 6 h. Cells were stained with a Zombie Aqua™ Fixable Viability Kit to label the dead cells, and their Fc receptor was blocked with TruStain FcX™ PLUS. After the surface staining with antibodies against CD45, CD11b, CD3e, CD19, MHCII (strategy 1, for general analysis); or CD45, EpCAM, CD31, Sca-1, Thy-1.2 (strategy 2, for tissue component cells); or CD11b, CD11c, CD86, CD206, MHCII (strategy 3, for antigen-presenting myeloid cells); or CD11b, CD115, MHCII, Ly-6G, c-kit (strategy 4, for non-antigen-presenting myeloid cells); or CD3e, CD4, ST2 (strategy 5, for T cells) diluted in True-Stain Monocyte Blocker™ and 2% FBS/PBS, cells were fixed with FluoroFix™ Buffer. Then cells were permeabilized and stained with antibodies according to the strategy:
1. Strategy 1: cells were stained with antibodies against IL-6, RANKL, IL-10, IL-17A, ST2, or IL-33 diluted in Intracellular Staining Permeabilization Wash Buffer, except for mST2 examination (antibody against ST2 was added in surface staining when examine mST2).
2. Strategy 2: cells were stained with antibodies against IL-6, RANKL, ST2, or IL-33 diluted in Intracellular Staining Permeabilization Wash Buffer.
3. Strategy 3/4: cells were stained with antibodies against IL-6, RANKL diluted in Intracellular Staining Permeabilization Wash Buffer.
4. Strategy 5: cells were sequentially permeabilized with fixation diluent and permeabilization buffer in True-Nuclear Transcription Factor Buffer Set (Biolegend), and stained with antibodies against cytokines and transcription factors diluted in the permeabilization buffer.

For staining cells from primary cell culture:
Live cells in each sample were counted with Trypan blue staining, and at least 200,000 cells were kept for the staining. Cells were stained with a Zombie Aqua™ Fixable Viability Kit to label the dead cells, their Fc receptor was blocked with TruStain FcX™ PLUS, and then surface stained with antibodies against CD11b, F4/80, CD206 diluted in True-Stain Monocyte Blocker™ and 2% FBS/PBS.

Cells were filtered with a 35 µm cell strainer before loading to flow cytometry.

Instrument	CytoFLEX S (Beckman Coulter)
Software	CytExpert v2.3.1.22 (Beckman Coulter) and Kaluza v2.1 (Beckman Coulter)
Cell population abundance	Cells were only analyzed and not sorted. Cell population abundance were presented as percentages in Supplementary Figs.

Gating strategy

Cells obtained from tissue digestion were identified using FCS/SSC gate, and the doublets were excluded using FSC/FSC-width gate. After re-selecting the live cells with Zombie Aqua™ negative gate, cells were analyzed using five strategies below:
Strategy 1:
Immune-related and hematopoietic cells were defined as CD45+ cells; myeloid cells were defined as CD45+CD11b+ cells; T cells were defined as CD45+CD3e+ cells; B cells were defined as CD45+CD19+ cells; antigen presenting cells were defined as MHCII+ cells; antigen presenting myeloid cells were defined as CD45+CD11b+MHCII+ cells; non-antigen presenting myeloid cells were defined as CD45+CD11b+MHCII– cells; tissue-component cells were defined as CD45– cells.
Strategy 2:
Tissue-component cells were defined as CD45– cells; epithelial cells were defined as CD45–EpCAM+ cells; endothelial cells were defined as CD45–EpCAM–CD31+ cells; endothelial cells were defined as CD45–EpCAM–CD31+ cells; stem cells were defined as CD45–EpCAM–CD31– Sca-1+ cells; fibroblast/stromal cells were defined as CD45–EpCAM–CD31–Sca-1– cells. Fibroblast/stromal cells were sub-clustered by Thy-1.2 positive or negative.
Strategy 3:
Myeloid cells were defined as CD11b+ cells; dendritic cells were defined as CD11b+CD11c+MHCII+ cells; M1 macrophages were defined as CD11b+CD11c–MHCII+CD86+CD206– cells; M2 macrophages were defined as CD11b+CD11c–MHCII–CD86–CD206+ cells; periodontal tissue-resident macrophages were defined as CD11b+CD11c–MHCII+CD86–CD206+ cells.
Strategy 4:
Myeloid cells were defined as CD11b+ cells; monocytes were defined as CD11b+MHCII–CD115+ cells; neutrophils were defined as CD11b+MHCII–CD115–Ly-6G+ cells; mast cells were defined as CD11b+MHCII–CD115–Ly-6G–c-kit+ cells.
Strategy 5:
T cells were defined as CD3e+ cells; Th cells were defined as CD3e+CD4+ cells.
Five strategies were performed with different samples and the boundaries of positive or negative were defined using non-

staining and isotype controls of corresponding tissues. Compensation for each strategy with different cytokines were performed using cells from corresponding tissues for Zombie Aqua and UltraComp eBeads™ Compensation Beads for antibodies.

Cells obtained from macrophage polarization were identified using FCS/SSC gate, and the doublets were excluded using FSC/FSC-width gate. After re-selecting the live cells with Zombie Aqua™ negative gate, CD11b+F4/80+ double positive cells were defined as macrophages, and the expression of CD206 was analyzed. Positive gates were defined using non-staining and isotype control of M0 macrophages, and the compensation were performed using cells for Zombie Aqua™ and UltraComp eBeads™ Compensation Beads for antibodies.

☒ Tick this box to confirm that a figure exemplifying the gating strategy is provided in the Supplementary Information.
